# Integrated SNP and SV Analyses Reveal Genetic Mechanisms Underlying High-Altitude Adaptation in Goats

**DOI:** 10.3390/ani16142177

**Published:** 2026-07-13

**Authors:** Wenze Li, Yixin Su, Can Liu, Xiaokun Lin, Shanhui Xue, Bouabid Badaoui, Xiaochun Yan, Qi Lv, Rui Su

**Affiliations:** 1 College of Animal Science, Inner Mongolia Agricultural University, Hohhot 010018, China; 18394317062@163.com (W.L.); yixinwink@126.com (Y.S.); 15847756571@163.com (C.L.); 17860727391@163.com (X.L.); nndxsh@163.com (S.X.); yanxiaochunan@163.com (X.Y.); lvqi1202@imau.edu.cn (Q.L.); 2 Sino-Arabian Joint Laboratory of Sheep and Goat Germplasm Innovation, Hohhot 010018, China; 3 Inner Mongolia Key Laboratory of Sheep & Goat Genetics Breeding and Reproduction, Hohhot 010018, China; 4 Laboratory of Biodiversity, Ecology and Genome, Department of Biology, Faculty of Sciences Rabat, Mohammed V University, Rabat 10106, Morocco; bouabidbadaoui@gmail.com

**Keywords:** goat, structural variation, altitude adaptation, selection signature

## Abstract

High-altitude environments, characterized by hypoxia, intense ultraviolet radiation, and low temperatures, pose major challenges to livestock survival. In recent years, researchers have gradually uncovered adaptive mechanisms in livestock across different altitudes using whole-genome resequencing. Previous studies of goat altitude adaptation have been limited by small breed numbers and low sequencing depth, hindering comprehensive exploration of adaptive mechanisms across different altitudes. This study analyzed whole-genome resequencing data from 151 individuals across 17 goat breeds representing three distinct altitude gradients (high, middle, and low). Using both SNPs and structural variations (SVs), we characterized population relationships, gene flow, and the SV landscape, including QTL–SV associations and transposable element interactions. Selective sweep analyses using F_ST_, θπ ratio, XP-CLR, XP-EHH, and LFMM identified several candidate genes associated with altitude adaptation, including *ABCC4*, *RPS6*, *DSG4*, and *LY9*, which were significantly enriched in pathways related to hypoxia response, oxidative stress, energy metabolism, angiogenesis, and nervous system regulation. Notably, *ABCC4* showed ABCC4 showed recurrent candidate selection signals in both SNP and SV analyses, suggesting its potential involvement in altitude adaptation. These findings provide multi-level genomic evidence for goat adaptation to high-altitude stress and provide important insights into the adaptive evolution of goats.

## Simple Summary

High-altitude environments, characterized by low oxygen, strong ultraviolet radiation, and low temperatures, impose strong challenges on livestock survival and production. Although previous studies have explored altitude adaptation in goats, limited breed representation and sequencing depth have restricted a comprehensive understanding of the genetic mechanisms involved. In this study, we analyzed whole-genome sequencing data from 151 goats representing 17 Chinese breeds distributed across high-altitude, middle-altitude, and low-altitude regions. By integrating SNPs and structural variations (SVs) in the genome, we characterized population relationships, gene flow patterns, and genomic variation across different altitude groups. We further identified several candidate genes and biological pathways that may contribute to altitude adaptation, particularly those related to oxygen response, oxidative stress, energy metabolism, blood vessel regulation, and nervous system function. Among these, genes such as *ABCC4*, *RPS6*, *DSG4*, and *LY9* showed important signals associated with environmental adaptation. These findings provide a more comprehensive view of how goats may genetically adapt to high-altitude environments and offer useful information for the conservation, evaluation, and genetic improvement of goat breeds living in challenging ecological regions.

## 1. Introduction

Goats (*Capra hircus*) were among the earliest domesticated livestock species, with domestication dating back to the early Neolithic period (ca. 11,000 YBP) in the Fertile Crescent [1,2]. Depending on the economic traits required by humans, goats have gradually developed breeds with different traits (meat, dairy, wool, cashmere and leather) over a long period of artificial selection [3]. According to the 2024 National Catalogue of Animal Genetic Resources of China, there are 90 domestic goat breeds, including 69 indigenous breeds, 15 developed breeds and 6 introduced breeds. These breeds are widely distributed across all regions of China, providing meat, milk, fiber, and other livestock products, and represent a critical source of income for pastoralists. China has a vast territory with dramatic geographic, altitudinal, and ecological heterogeneity, with elevations ranging from −154.31 m to 8848.86 m. These diverse environmental conditions have promoted genetic diversity and adaptive evolution in goats. Notably, cashmere goats are widely distributed across middle- and high-altitude regions such as plateaus and mountainous areas, and exhibit strong adaptation to local stressors including low temperature, hypobaric hypoxia, and high ultraviolet radiation. Understanding the genetic basis of high-altitude adaptation in goats is crucial for elucidating evolutionary mechanisms and optimizing breeding strategies to improve livestock resilience.

Adaptation is a core process in biological evolution, through which species acquire traits that improve fitness in local environments, thereby enhancing the transmission of genetic information across generations. Extreme environmental pressures are widely recognized as key drivers in shaping animal genomes [4]. Accordingly, population genomics has emerged as the primary means of investigating the evolutionary mechanisms by which mammals adapt to their environments. High-altitude environments present significant challenges for animals, including hypoxia, low temperatures, and intense ultraviolet radiation. These factors, alongside climatic variables such as light exposure and precipitation, collectively shape complex environmental selection pressures, thereby driving the emergence of specific adaptive phenotypes in animal species [5]. Exposure to high altitude alters aerobic metabolic pathways and disrupts oxygen homeostasis in mammals, impacting core physiological processes including energy metabolism, hemodynamics, and cellular redox regulation [6]. This also indicates that high-altitude adaptation is a complex process involving multi-factor regulation encompassing morphology, physiology, biochemistry, and host-associated microbiota. Moreover, extensive genomic studies reveal that numerous domesticated plateau animals achieve rapid colonization and adaptation to high-altitude environments not only through simple lowland dispersal but also through natural selection and the process of introgression from closely related species [7].

Selective sweep analysis is pivotal for elucidating the genetic mechanisms underlying altitudinal adaptation in mammals. Natural selection elevates the frequency of adaptive variation within populations, thereby shaping specific genomic signatures. These variations can be identified through genome-wide selective sweep methods. Recently, the rapid development of high-throughput sequencing technologies, combined with population genetics methods (e.g., F_ST_, XP-EHH, and XP-CLR), has enabled researchers to systematically identify candidate genes and functional pathways associated with high-altitude adaptation. Investigations into altitudinal adaptation have been conducted in species such as sheep [8,9,10], pigs [11,12,13], cattle [14,15], yak [16,17], and horses [18,19]. Although related studies have also been performed on goats from high-altitude regions [9,20], the number of breeds investigated is limited, and studies utilizing middle-altitude goats as experimental controls are lacking.

This study analyzed 151 individuals from 17 Chinese goat breeds, which were stratified into low-altitude (<1000 m), middle-altitude (1000–4000 m), and high-altitude (>4000 m) groups based on their environmental altitude. We used whole-genome resequencing data to compare genomic differentiation among these altitude groups and to identify candidate genes for altitude adaptation using selective sweep analysis. These findings provide an empirical foundation for further elucidating the molecular regulatory mechanisms of altitudinal adaptation in goats.

## 2. Materials and Methods

### 2.1. Data Records

**Figure 1:**
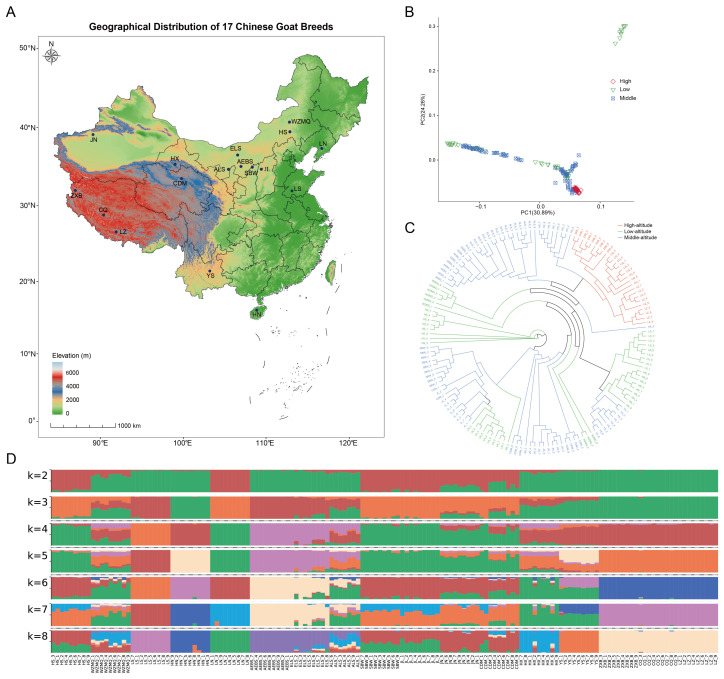
Geographical distribution and population structure of 17 domestic goat breeds. (**A**) Sampling locations and altitude conditions of 17 goat breeds. (**B**) Principal component analysis (PCA) of goat populations from high, middle and low-altitude groups. Different colors and shapes represent distinct altitude groups. (**C**) Phylogenetic tree of 151 goats constructed from genome-wide SNPs. (**D**) Genetic structure of 17 goat populations. K denotes the number of putative ancestral populations. Different colors within each vertical bar represent populations potentially derived from distinct ancestors.

Tissue samples were collected from 151 goats across 11 provinces in China, spanning a wide altitudinal gradient, as part of prior work (Figure 1A). Genomic DNA was extracted from each individual and paired-end sequencing libraries were constructed. Paired-end 150 bp sequencing was performed on an MGISEQ2000 platform (MGI Tech Co., Ltd., Shenzhen, China). Based on the altitude of the sampling sites, the goat populations were classified into low-altitude (<1000 m, *n* = 45), middle-altitude (1000–4000 m, *n* = 79), and high-altitude (>4000 m, *n* = 27) populations. These thresholds were used as operational categories to represent broad environmental gradients in the available sampling design: low-altitude populations represented environments with relatively limited hypobaric stress, high-altitude populations represented environments with pronounced hypobaric hypoxia and associated climatic stressors, and the middle-altitude group served as an intermediate comparison category. This classification was based on the distribution of the available sampling sites and was not intended to define physiologically homogeneous altitude classes. Therefore, the middle-altitude group should be interpreted as an intermediate altitude-related category rather than as a uniform moderate-hypoxia population.

### 2.2. Sequence Alignment

We filtered raw reads using fastp v0.23.2 [21]. Reads were removed if they met any of the following criteria: >10% unidentified nucleotides (N), presence of adapter sequences, or ≥50% of bases with Phred quality scores < 5. After filtering, we obtained 13.18 Tb of clean data.

The clean data were mapped to the cashmere goat reference genome (GCA_040822015.1) [22] using BWA v0.7.8 with the command ‘mem -t 100 -k 32 -M’, where -k specifies the minimum seed length and -M marks shorter split alignments as secondary [23]. To reduce mismatch generated by PCR duplicates during library preparation, duplicate reads were marked and removed using Picard v3.1.0 (https://broadinstitute.github.io/picard/, accessed on 20 May 2025). After duplicate removal, an average of 99.87% of the reads were successfully mapped to the reference genome. The mean sequencing coverage per individual was 30.40×.

### 2.3. SNP Calling

SNPs were called using GATK v3.6 [24]. Briefly, the HaplotypeCaller module was employed for SNP discovery, followed by joint genotyping with the CombineGVCFs module, and separation of SNPs from indels using the SelectVariants module. The SNP dataset was then filtered using the VariantFiltration module. Filtering thresholds were set as follows: QUAL < 30, QD < 2.0, FS > 60.0, MQ < 40.0, SOR > 3.0, and MQRankSum < −12.5. Further filtering was performed on the SNP dataset using VCFtools v0.1.16 [25] with the parameters: -maf 0.05 -max-missing 0.9 -hwe 1 × 10^−6^. This process yielded a final set of 14,973,532 high-quality SNPs for subsequent analyses.

### 2.4. Structural Variation (SV) Calling and Annotation

To ensure high accuracy of the SV dataset, we adopted an integrated strategy to detect SVs from short-read alignments. SVs were detected in each sample using five independent tools: Delly v1.2.6 [26], Lumpy v0.3.1 [27], Dysgu v1.8.0 [28], Manta v1.6.0 [29], and Wham v1.8.0 [30]. All tools were run with default parameters for SV detection. To reduce the false-positive rate, the SV calls from each tool were filtered and then merged. Merging across all samples was performed using SURVIVOR v1.0.6 [31] with the command “SURVIVOR merge sample.txt 1000 3 1 1 0 50 all_sample.vcf”. Only the SVs ≤ 1 Mb that were detected by at least three tools were retained to improve specificity and reduce false positives inherent in short-read SV detection. Because insertions (INSs) are more difficult to detect accurately from short-read sequencing data than deletions (DELs) or duplications (DUPs), particularly in repetitive genomic regions, the multi-caller consensus strategy was used to reduce the likelihood of false-positive INS calls relative to single-caller results. However, this strategy does not constitute experimental validation and cannot completely eliminate false positives. Finally, the merged SVs were genotyped using Graphtyper v2.7.7 [32]. SVs were annotated with ANNOVAR v2020-06-08 [33] and were categorized into nine functional categories: intergenic region, intronic region, exonic region, downstream region, upstream region, upstream/downstream regions, 5′ UTR, 3′ UTR, and splicing sites.

To investigate the potential association of transposable elements (TEs) with SVs, we analyzed SV-TE overlaps using the detected SV dataset. Based on the TE annotation file from our previously assembled Inner Mongolia cashmere goat reference genome [22], overlaps between SVs and TEs were identified using BEDTools v2.30.0 [34]. Notably, we investigated the overlap pattern between SVs ≤ 10 kb in length and TEs.

### 2.5. Population Structure and Phylogenetic Analysis

A genetic distance matrix was calculated using PLINK v1.90 [35] (using the --distance-matrix option), and a neighbor-joining (NJ) phylogenetic tree was constructed with Phylip v.3.696 (https://github.com/topics/phylip/, accessed on 15 June 2025) [36]. Tree visualization was performed using FigTree v1.4.4 (https://tree.bio.ed.ac.uk/software/figtree/, accessed on 15 June 2025). Principal component analysis (PCA) was performed using the PLINK v1.90, and visualization was conducted using the ggplot2 package in R v4.3. ADMIXTURE v1.3.0 [37] was used to perform population structure analysis for K values ranging from 2 to 10. The optimal K was chosen as the value with the lowest cross-validation (CV) error.

### 2.6. General Genomic Characteristics

We used PLINK v1.90 [35] to calculate observed heterozygosity (*H*o), expected heterozygosity (*H*e), and the inbreeding coefficient (F_ROH_) to assess the genetic diversity of the goat populations. Runs of Homozygosity (ROH) detection was performed with PLINK v1.90 using the following parameters: “-homozyg-window-snp 50 --homozyg-snp 50 --homozyg-kb 200 --homozyg-density 50 --homozyg-gap 100 --homozyg-window-missing 2 --homozyg-window-threshold 0.01 --homozyg-window-het 0” [38]. After ROH identification, the number and total length of ROHs were calculated for each breed, and the ROHs were classified by length into five classes: 0–0.5 Mb, 0.5–1 Mb, 1–2 Mb, 2–4 Mb, and >4 Mb.

Treemix v1.12 [39] was used to infer gene flow among breeds based on allele frequency data. To evaluate the effect of different numbers of migration events (m) on model fit, we ran multiple models with m = 1–13. Each migration number was replicated five times to ensure the stability of the results. The optimal migration model was determined when the explained variance reached 99.8%. Finally, a maximum-likelihood tree was constructed using *Capra ibex* (NCBI Sequence Read Archive (SRA) accession: SRR5260690) as the outgroup. Gene flow between populations was visually represented as directional arrows on the tree.

The linkage disequilibrium (LD) decay rate for each breed was calculated based on SNP data using PopLDdecay v3.40 [40]. PLINK v1.90 was used to estimate pairwise LD between SVs within each chromosome and to assess the LD between SVs and SNPs within a 50 kb window. Specific parameters, adapted from a previous study [41], were set as “-ld-window 99999 -ld-window-kb 1000 -ld-window-r2 0”.

### 2.7. Identification of SV Hotspots and Overlap with QTL Regions

Using a sliding window approach (200 kb window, 100 kb step size), we quantified SV breakpoint counts along each chromosome and ranked windows by their breakpoint density. The top 10% of windows by SV breakpoint density were defined as SV hotspots, following previous SV studies [42]. The terminal 5 Mb regions of each chromosome arm were defined as telomeric regions [43]. We compared the distribution of SV breakpoints between telomeric and non-telomeric regions, with statistical significance assessed using the Wilcoxon rank-sum test. Furthermore, we implemented a Python v3.9.21 to compare the identified SV hotspots with quantitative trait loci (QTL) in the Animal QTL Database (https://www.animalgenome.org/cgi-bin/QTLdb/index/, accessed on 21 July 2025).

### 2.8. Selective Sweep Analysis During Environmental Adaptation

The pairwise fixation index (F_ST_) and θπ ratio (π_case_/π_control_) were calculated using VCFtools v0.1.16 [25] with 50 kb sliding windows with a 20 kb step size for high-altitude, middle-altitude, and low-altitude groups. The cross population composite likelihood ratio (XP-CLR) is a method for the determination of the selection signal, mainly used to detect gene frequency changes caused by natural selection among different populations. It is possible to identify gene regions that may be under strong natural selection pressure between two populations. Inter-population analysis was performed using xpclr v1.1.2 [44] with a 50 kb non-overlapping sliding window, each window containing up to 200 SNP sites. Cross-population extended haplotype homozygosity (XP-EHH) was used in the calculation of recently occurring selection signals between two populations using linkage disequilibrium. XP-EHH values between two populations were calculated using selscan v2.0.0 [45] and normalized against the mean value of XPEHH in a 50 kb region.

#### Rationale for Empirical Outlier Threshold

The top 1% of genomic windows from each selection-scan statistic were used as an empirical outlier threshold to define candidate selection signals, rather than as a formal statistical significance threshold. This threshold was adopted for three reasons. First, F_ST_, θπ ratio, XP-CLR, and XP-EHH capture different features of selection and generate statistics with different scales and distributions, making a single unified parametric significance threshold unsuitable across methods. Second, in population genomic studies based on whole-genome resequencing data, empirical top-ranking windows are commonly used to identify outlier regions for downstream candidate-gene screening. Third, the top 1% cutoff provides a conservative preliminary filter that retains the strongest empirical signals while limiting the number of candidate windows for cross-method comparison. Previous studies using analogous livestock genomic datasets, including Xizang sheep, dairy goats, BoHuai goats, Tianzhu White Yak, and Dianzhong cattle, have applied the top 1% genomic windows or variants to identify candidate regions under selection [6,46,47,48,49].

The use of a top 1% cutoff may retain false-positive signals, particularly in genomic regions affected by demographic history, population structure, genetic drift, local recombination variation, or method-specific statistical bias. To reduce false-positive interpretation from any single method and improve reproducibility, candidate regions were prioritized using a hierarchical procedure. First, for each pairwise altitude comparison, genome-wide windows were ranked separately according to each selection statistic, including F_ST_, θπ ratio, XP-CLR, and XP-EHH. Second, the top 1% windows from each statistic were retained as preliminary candidate windows. Third, genes overlapping these candidate windows were annotated. Fourth, SNP-based candidate genes supported by at least three of the four selection-scan methods were retained as high-confidence candidate genes for each pairwise comparison. Finally, genes repeatedly detected across different pairwise comparisons or supported by both SNP- and SV-based analyses were considered higher-priority candidates for biological interpretation. SV-based F_ST_ outliers and LFMM-based altitude-associated SVs were used as complementary evidence. This prioritization strategy was designed to reduce overinterpretation of signals detected by a single method or marker type.

This conservative intersection-based strategy may also increase the risk of false-negative results. True adaptive loci may be missed if they show weak selection signals, reflect polygenic adaptation, fall near window boundaries, are detectable by only one or two selection statistics, or do not rank within the top 1% in at least three methods. Therefore, the candidate regions reported in this study should be interpreted as a conservative set of high-confidence putative selection regions rather than a complete catalog of all loci involved in altitude adaptation.

### 2.9. SV-Based Genome-Wide Environmental Association Study (GWEAS)

Altitude data for the sampling locations of 17 goat populations were collected, and a genome-wide environmental association study (GWEAS) was performed on 53,500 SVs from 151 goats using the latent factor mixed model (LFMM). To reduce the potential confounding effects of population structure and breed history, LFMM was used to test associations between SV genotypes and altitude while accounting for latent genetic structure. The SV VCF file was converted into a genotype file using VCFtools v0.1.16 [25]. Using the LFMM v2 package [50] in R, the genomic inflation factor (λ) was calculated for K = 1–10. The K value (K = 3) at which the genomic inflation factor (λ) was closest to 1 was selected as the optimal number of latent factors for GWEAS. Candidate SVs associated with the altitude variable were identified using a threshold of adjusted *p*-value (*p*-adj) < 0.05 from the LFMM results, followed by gene annotation [41].

### 2.10. Gene Annotation and Enrichment Analysis

The cashmere goat reference assembly was used, and candidate genes were identified by gene annotation of the selected regions using BEDtools v2.30.0 [34]. To investigate gene function and potential regulatory mechanisms, we performed functional enrichment analysis. Functional enrichment was performed using KOBAS web server (http://bioinfo.org/kobas/, accessed on 9 October 2025). Enrichment for Gene Ontology (GO) terms and Kyoto Encyclopedia of Genes and Genomes (KEGG) pathways was tested using the hypergeometric test. GO terms and KEGG pathways with *p*-value < 0.05 were considered enriched and were used for functional interpretation. To explain the potential impacts of these genes on livestock performance, we reviewed the literature and public databases (e.g., NCBI PubMed) for reported functional effects of candidate genes.

## 3. Results

### 3.1. Detection of Genome-Wide SNPs and Population Structure

In this study, we collected whole-genome resequencing data from 151 individuals representing 17 Chinese goat breeds, originally collected in the team’s prior research (Figure 1A). These 151 goat genomes were grouped by sampling altitude as follows: high-altitude goats (>4000 m; *n* = 27), low-altitude goats (<1000 m; *n* = 45), and middle-altitude goats (1000–4000 m; *n* = 79). The total dataset comprised ~3.54 Tb of sequence data, with an average sequencing depth of 30.40× per individual (Table S1). To ensure data quality for downstream analyses, raw reads were subjected to stringent quality control and filtering, yielding a final set of 14,973,532 high-quality SNPs.

Principal component analysis (PCA) was performed on the filtered SNP dataset. The first two principal components (PC1 and PC2) explained 30.89% and 24.26% of the total genetic variance, respectively (Figure 1B). High-altitude and low-altitude breeds formed distinct clusters, whereas middle-altitude populations showed partial overlap with both groups, indicating that genetic divergence among the 17 goat populations was associated with both altitude-related geography and breed history. In addition, domestic goat breeds clustered according to their breeding history (Figure S1). Phylogenetic tree analysis further reinforced the PCA results (Figure 1C), with closely related breeds forming distinct clades. The two meat-purpose breeds and the dairy breed clustered into a separate branch, showing clear divergence from the cashmere goats.

To further resolve genetic divergence among the 17 goat breeds, ancestry proportions were estimated using ADMIXTURE. The cross-validation error was minimized at K = 3 (CV error = 0.37471), indicating that the ancestral structure of the 17 breeds is best represented by three ancestral components (Figure 1D). Breeds such as Hainan black goat (HN), Yunshang black goat (YS), and Xizang cashmere goats (ZXB, CQ, LZ) shared one ancestral component. Inner Mongolian cashmere goats (AEBS, ALS, ELS) shared another with Laoshan dairy goats (LS), while Liaoning cashmere goats (LN) and their derived breeds shared a third predominant ancestry. This pattern is consistent with the phylogenetic tree. Furthermore, Alashan cashmere goat (ALS), Hexi cashmere goat (HX) and Vjumqin white goat (WZMQ) displayed admixed ancestral compositions, suggesting historical gene flow from multiple sources during their selection history.

### 3.2. Patterns of Genomic Diversity

**Figure 2:**
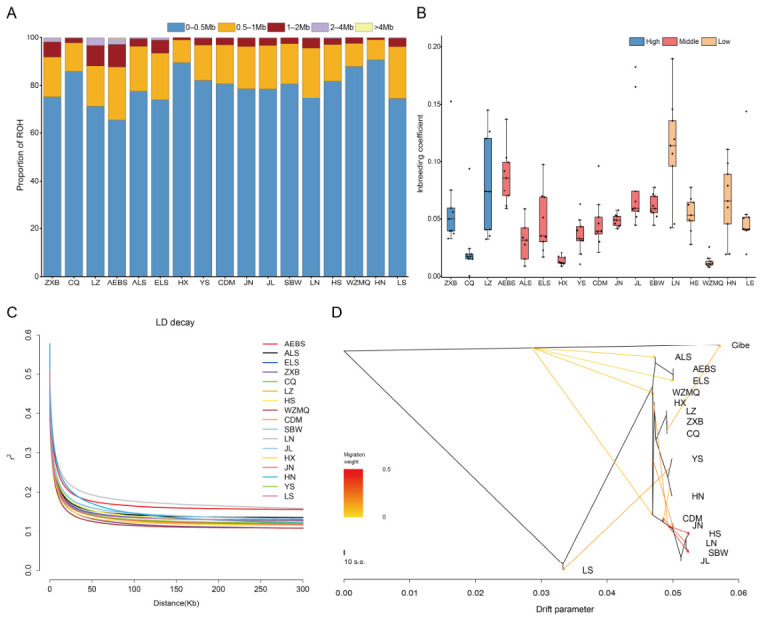
Analysis of genetic diversity in domestic goat populations. (**A**) Proportion of runs of homozygosity (ROH) by length class across the genome for each breed. (**B**) Mean inbreeding coefficient (F_ROH_) for each breed. The black line in the boxplot represents the median line, and outliers are shown as individual points. (**C**) Linkage disequilibrium (LD) decay curves based on SNPs. (**D**) Gene flows among goat populations, with 99.8% of genetic variance explained at m = 12.

To further evaluate genetic diversity among domestic goat breeds, we calculated the expected heterozygosity (*H*e), observed heterozygosity (*H*o), and the inbreeding coefficient (F_ROH_) for each breed based on the SNP dataset. The distribution patterns of runs of homozygosity (ROH) revealed breed-specific differences in ROH length distributions. Populations including LN and AEBS harbored a higher proportion of long ROHs (>4 Mb), indicative of recent inbreeding events, whereas populations such as WZMQ and ZXB were dominated by short ROHs (0.5–1 Mb), suggesting more diverse genetic backgrounds (Figure 2A). The F_ROH_ calculated from ROHs further corroborated this trend (Figure 2B). The LN breed exhibited the highest inbreeding coefficient (F_ROH_ = 0.1106), followed by AEBS (F_ROH_ = 0.0870), while the WZMQ breed had the lowest level of inbreeding (F_ROH_ = 0.0126) (Table S2). These results are consistent with the ROH fragment distribution patterns. Ho was slightly higher than He across all goat populations. This indicates a mild heterozygote excess within the populations, potentially attributable to gene flow between groups or hybrid breeding (Table S3).

Additionally, we performed linkage disequilibrium (LD) decay analysis for all goat populations to assess their genetic diversity and selection history. Distinct LD decay rates were observed among breeds (Figure 2C). The slowest LD decay was observed in the LN population, followed by AEBS and HN, whereas WZMQ and ZXB exhibited the most rapid decay. Generally, a slower LD decay rate is associated with lower effective population size and stronger selection pressure. These results suggest that the ZXB and WZMQ populations have experienced less intense selection and maintain higher genetic diversity than other breeds.

To infer historical gene flow events among Chinese goat breeds, we analyzed the 17 breeds using TreeMix with the *Capra ibex* as an outgroup. By evaluating the model fit under different numbers of migration events (m), we identified m = 12 as the optimal model, where the proportion of explained genetic variance exceeded 99.8% and the likelihood value stabilized (Figure S2). The maximum-likelihood population tree constructed under the optimal model (m = 12) showed that populations generally clustered according to geographical distribution (Figure 2D). This result is consistent with the SV-based phylogenetic tree generated. Overall, populations were divided into several major clades, such as the Inner Mongolian group (AEBS, ELS, ALS) and the Xizang group (ZXB, CQ, LZ). Notably, the Liaoning cashmere goat and its derived breeds (CDM, JN, HS, SBW, JL) formed a distinct clade and exhibited strong gene flow characterized by high migration weights. This pattern aligns with their documented breeding history. Furthermore, signals of gene flow were detected between the CQ and Capra ibex, whereas gene flow events were not found between other goats from this region and other domestic breeds, indicating genetic distinctiveness of the breeds from this region. This pattern may reflect the genetic distinctiveness of goats from this region, potentially associated with their geographic isolation and adaptation to high-altitude environments (Figure 2D).

### 3.3. Discovery and Characterization of Structural Variations in Goats

**Figure 3:**
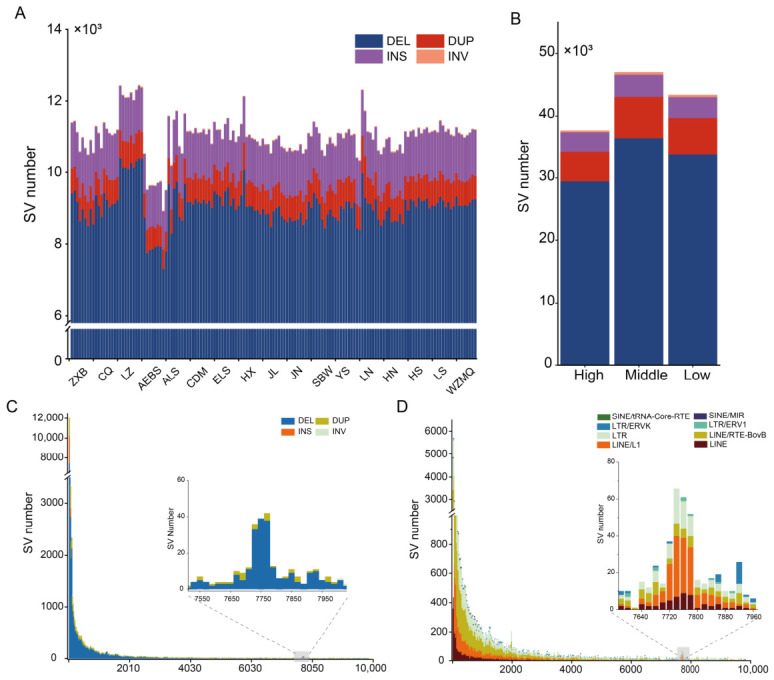
Distribution characteristics of SVs in goats. (**A**) Number of SVs in 151 goat individuals. Four SV types are deletion (DEL), duplication (DUP), insertion (INS), and inversion (INV). (**B**) Average number of SVs per population for high-altitude, middle-altitude, and low-altitude groups. (**C**) Distribution of SVs by length of 1–10,000 bp in goat populations. (**D**) Distribution of transposable elements (TEs) by length of 1–10,000 bp in goat populations.

To enhance the accuracy of structural variation (SV) detection, we employed five high-confidence SV tools: Delly, Lumpy, Dysgu, Manta, and Wham. A total of 53,500 high-confidence SVs were identified, with an average of 11,014 SVs per individual (Figure 3A). These SVs consisted of 41,106 DELs, 3911 INSs, 8009 DUPs, and 474 inversions (INVs). The middle-altitude group harbored the highest number of SVs (47,051), surpassing the high-altitude (37,602) and low-altitude (43,354) groups (Figure 3B; Table S4). Genomic annotation indicated that 66.79% were located in intergenic regions, while 30.21% were intronic. A total of 454 SVs (0.85%) were found in exonic regions (Figure S3). The SV frequency spectrum was skewed towards rare alleles, with 14,008 SVs (26.18%) exhibiting a minor allele frequency (MAF) of less than 0.01 (Table S5).

The lengths of all SV types were quantified. Most SVs (58.81% of the total) were relatively short, ranging from 50 bp to 500 bp. Notably, 99.94% of INSs fell within the 50–500 bp range. This observation is primarily attributable to the inherent limitations of short-read-based SV tools in precisely resolving INS events. Furthermore, a prominent peak in SV length distribution between 100 and 150 bp was evident for both INSs and DELs (Figure 3C and Figure S4).

We investigated the association between SVs and TEs. The most abundant TE families associated with SVs in the goat genome were the RTE-BovB and L1 of LINEs (long interspersed nuclear element), ERVL of LTRs (long terminal repeat), tRNA-Core-RTE and MIR of SINEs (short interspersed nuclear element) (Table S6). Previous studies have reported that transposons from the LINE/RTE-BovB and SINE/tRNA-Core-RTE families contribute to an enrichment of SVs in the 100–150 bp and 7500–8000 bp size ranges in livestock genomes [41]. This phenomenon was also observed in our study. In the goat genome, the distribution of both SVs and TE families revealed a notable increase in the number of SVs measuring 100–150 bp and 7500–8000 bp, potentially originating from the tRNA-Core-RTE and L1/RTE-BovB families, respectively (Figure 3C,D). This finding further supports the role of TEs as major drivers of SV in the goat genome.

### 3.4. Population Structure and Linkage Patterns Revealed by SVs

**Figure 4:**
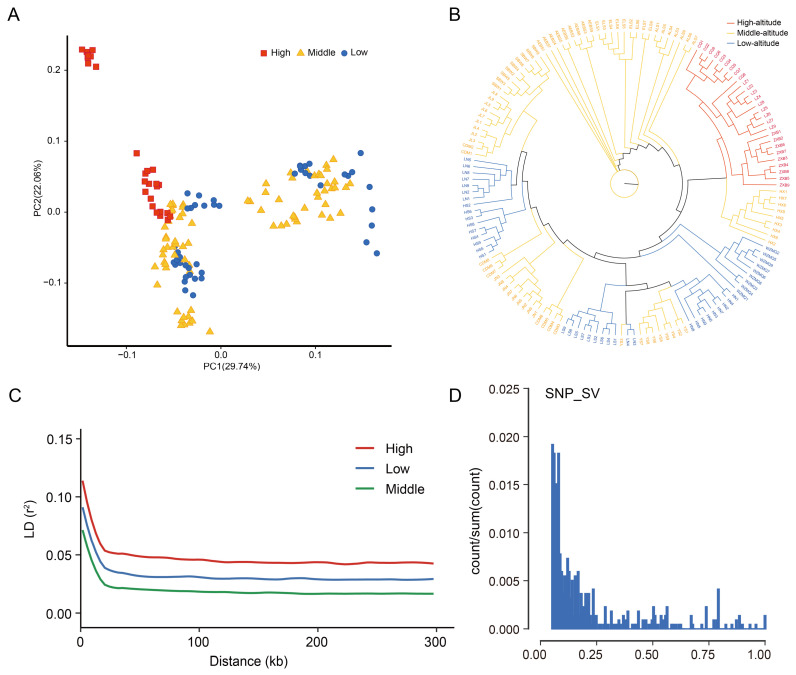
Population genetic analysis based on structural variations (SVs). (**A**) PCA of goats from different altitude groups based on genome-wide SVs. Principal component 1 (PC1) and principal component 2 (PC2) explained 29.74% and 22.06% of the total SV-derived genetic variation. Red squares denote high-altitude groups, yellow triangles denote middle-altitude groups, and blue circles denote low-altitude groups. (**B**) Neighbor-joining phylogenetic tree constructed using SVs, illustrating genetic relationships among goats from different altitude groups. (**C**) The pattern of linkage disequilibrium (LD) decay in goat genomes across different altitude groups. (**D**) LD distribution of SVs and their largest linked SNPs within a distance of 50 kb.

To evaluate whether SVs reveal genetic structure information distinct from SNPs, we performed population genetic structure analysis based on 53,500 high-quality SVs (Figure 4A). PCA based on SVs was largely concordant with SNP-based clustering and clearly distinguished high-altitude and low-altitude populations. However, compared to SNP results, PC1 of SV data provided clearer separation between high-altitude and middle-altitude populations. This observation was further supported by the phylogenetic tree from SVs (Figure 4B). Ancestry component inference analysis based on SVs was generally consistent with SNP-based results, though some populations (e.g., ZXB and CQ) exhibited more complex ancestral compositions under the SV model. These findings indicate that SVs capture dimensions of genetic variation not fully tagged by SNPs, highlighting the importance of integrating SVs into population genomic studies.

To further investigate the impact of environmental adaptation on goat genetic structure, we performed LD analysis across different altitude groups (Figure 4C). The results revealed the slowest LD decay in high-altitude populations. This may reflect stronger selection pressures imposed by the high-altitude environment on local goats, limiting genetic recombination. Middle-altitude populations exhibited the most rapid LD decay, indicating more frequent gene flow and weaker environmental selection pressures. Overall, these findings indicate that the harsh high-altitude environment is associated with the slowest LD decay.

Additionally, we evaluated LD between SVs and neighboring SNPs within 50 kb windows (Figure 4D). Among 53,500 SVs, only 3.8% (2048) showed detectable LD (R^2^ > 0) with neighboring SNPs. The vast majority (93.64%) showed low LD levels (R^2^ ≤ 0.2), while moderate (0.2 < R^2^ < 0.8) and high LD (R^2^ ≥ 0.8) accounted for only 5.93% and 0.59% respectively. This indicates that SNP data alone cannot fully capture the genetic diversity in goats.

### 3.5. Distribution of SV Hotspots

By analyzing the genomic distribution of SVs using 200 kb windows, we identified 875 SV hotspots (defined as the top 10% of windows based on SV breakpoint density) across goat chromosomes, spanning 373 Mb, which is consistent with previous findings [41]. To investigate the potential functional roles of SVs in complex trait variation, we obtained information on known goat quantitative trait locus (QTLs) from the Animal QTL Database (https://www.animalgenome.org/cgi-bin/QTLdb/index/, accessed on 21 July 2025). Comparative analysis revealed 54 SV hotspots overlapping with 19 reported QTLs for traits including body height, body length, chest width, teat number, and milk fat percentage (Table S7). This suggests that SV hotspots may be associated with economically relevant traits in goats.

Furthermore, we quantified SV breakpoints within the terminal 5 Mb regions (telomeric regions) of each chromosome arm and assessed the significance of their enrichment using the Wilcoxon rank-sum test. We detected 17,864 SV breakpoints (16.87%) in the telomeric regions, with a total of 300 Mb, compared to 88,017 (83.13%) in non-telomeric regions across 29 autosomes and the X chromosome (Table S8). Statistical analysis confirmed a significant enrichment of SV breakpoints in telomeric regions (Wilcoxon rank-sum = 1375, *p*-value = 2.439 × 10^−5^).

### 3.6. Genome-Wide SNP-Based Selective Sweeps Associated with Altitude Adaptation

#### 3.6.1. Selective Signatures of High-Altitude and Low-Altitude Populations

**Figure 5:**
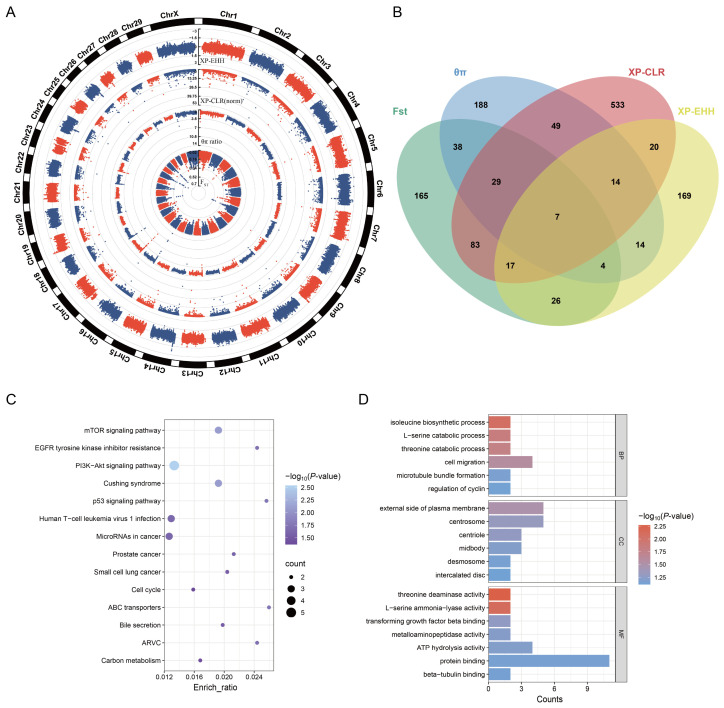
Selective signatures of high-altitude and low-altitude populations. (**A**) Selective sweeps of SNPs in high-altitude goats were analyzed using F_ST_, θπ, XP-CLR and XP-EHH methods. Regions in the top 1% of each method were designated as empirical candidate regions. The high-altitude group was treated as the focal population and the low-altitude group as the reference population. (**B**) Venn diagram showing the overlap of candidate genes under selection identified by the four methods. (**C**) KEGG enrichment results for candidate genes under selection simultaneously identified by three methods. (**D**) GO enrichment analysis of candidate genes under selection.

To identify selection regions associated with environmental adaptation, we performed a selective sweep analysis using SNPs, comparing the high-altitude population to the low-altitude as a control (Figure 5A,B). The analysis revealed the highest F_ST_ peaks on chromosomes 3, 8, 24, 26, and X. The θπ ratio revealed strong selection signals on chromosomes 16, 17, 18, and X. XP-CLR detected signals on chromosomes 2, 5, 11, 13, and 26, whereas XP-EHH identified candidate signals on chromosomes 6, 24, and 26. The intersection of the top 1% windows identified by three methods identified 71 genes (F_ST_ > 0.172803; θπ ratio > 1.396271; XP-CLR > 1.595656; normalized XP-EHH > 0.867749). Functional enrichment analysis of these candidate genes revealed 14 significantly enriched KEGG pathways, including the PI3K-Akt signaling pathway (*PRLR*, *PTEN*, *ITGA4*, *RPS6*), mTOR signaling pathway (*RPS6*, *WDR24*, *PTEN*), ABC transporters (*ABCC4*, *ABCB1*), and Cell cycle (*CCNE2*, *CDKN2C*). These pathways are principally related to hypoxia adaptation, oxidative stress, and energy metabolism (Figure 5C). GO analysis revealed 11 GO terms crucial for metabolic regulation and cellular functions, such as threonine deaminase activity, cell migration, and external side of plasma membrane (Figure 5D). Furthermore, seven genes (*ABCB1*, *ABCC4*, *CA1*, *DSG4*, *LY9*, *PGM5*, and *RPS6*) were consistently identified by all four methods (Figure 5B).

#### 3.6.2. Selective Signatures of High-Altitude and Middle-Altitude Populations

**Figure 6:**
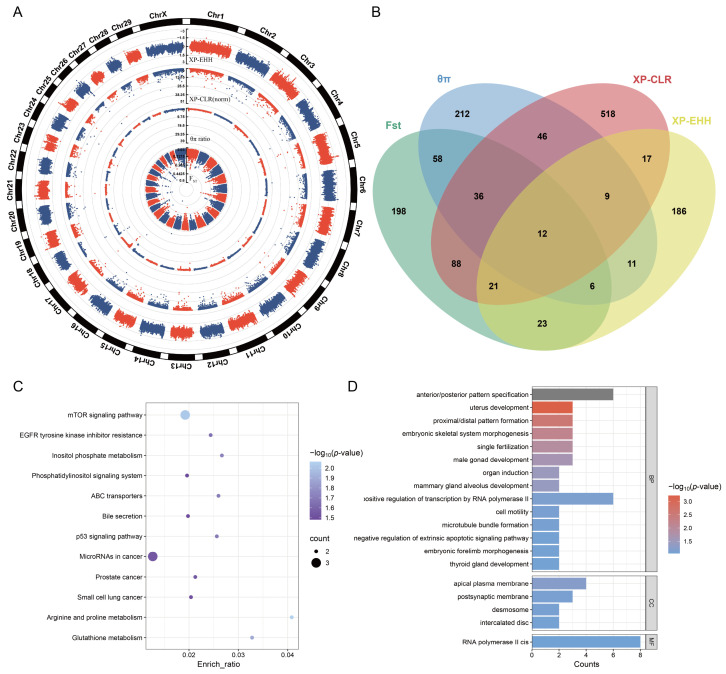
Selective signatures of high-altitude and middle-altitude populations. (**A**) Selective sweeps of SNPs in high-altitude goats were analyzed using F_ST_, θπ, XP-CLR and XP-EHH methods. Regions in the top 1% of each method were designated as empirical candidate regions. The high-altitude group was treated as the focal population and the middle-altitude group as the reference population. (**B**) Venn diagram showing the overlap of candidate genes under selection identified by the four methods. (**C**) KEGG enrichment results for candidate genes under selection simultaneously identified by three methods. (**D**) GO enrichment analysis of candidate genes under selection.

Using the same methodology, we performed a selective sweep analysis of the high-altitude population, with the middle-altitude population as the control (Figure 6A,B). Using the top 1% of windows as candidate signals, the F_ST_ analysis identified 1326 candidate selective regions, corresponding to 442 annotated genes (F_ST_ > 0.148412). The θπ ratio analysis detected 1344 selective regions, corresponding to 390 annotated genes (θπ ratio > 1.377081). XP-CLR identified 1321 selective regions, within which 747 genes were annotated (XP-CLR > 1.335791). After normalizing XP-EHH in 50 kb windows, XP-EHH detected 548 candidate regions encompassing 285 annotated genes (normalized XP-EHH > 0.899281). Among these, 84 genes were jointly identified by at least three methods. These candidate genes were significantly enriched in 12 KEGG pathways, including arginine and proline metabolism, the mTOR signaling pathway, glutathione metabolism, and ABC transporters, which are primarily associated with energy metabolism, oxidative stress response, and vascular regulation (Figure 6C). GO enrichment analysis further revealed significant enrichment in eight GO terms, such as embryonic skeletal system morphogenesis and anterior/posterior pattern specification (Figure 6D). In addition, twelve genes (*ABCC4*, *ARFGEF3*, *JMJD8*, *LY9*, *MCRIP2*, *METTL26*, *RHBDL1*, *RPS6*, *SLC17A5*, *TMEM62*, *WDR24*, and *WFIKKN1*) were consistently identified by all four methods (Figure 6B).

#### 3.6.3. Selective Signatures of Middle-Altitude and Low-Altitude Populations

**Figure 7:**
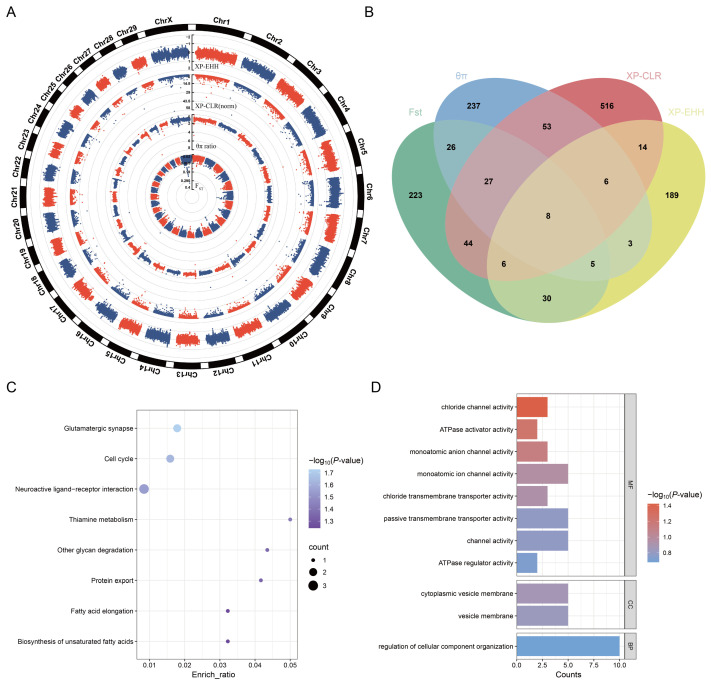
Selective signatures of middle-altitude and low-altitude populations. (**A**) Selective sweeps of SNPs in high-altitude goats were analyzed using F_ST_, θπ, XP-CLR and XP-EHH methods. Regions in the top 1% of each method were designated as empirical candidate regions. The middle-altitude group was treated as the focal population and the low-altitude group as the reference population. (**B**) Venn diagram showing the overlap of candidate genes under selection identified by the four methods. (**C**) KEGG enrichment results for candidate genes under selection simultaneously identified by three methods. (**D**) GO enrichment analysis of candidate genes under selection.

To further elucidate adaptive divergence across altitudinal gradients in goats, we conducted selective sweep analysis on middle-altitude populations using the low-altitude populations as the control. Using the top 1% of genomic windows as the candidate signals, the F_ST_, θπ, XP-CLR, and XP-EHH methods identified 369, 365, 674, and 261 candidate genes (Figure 7A) (FST > 0.054456; θπ ratio > 1.26097; normalized XP-CLR > 1.775511; normalized XP-EHH > 0.913793). A total of 52 genes were identified by three methods. Eight genes, *ABCC4*, *ANKRD65*, *ANO4*, *ATP8A1*, *CDKN2C*, *MRPL20*, *SLC15A2*, and *TMEM88B*, were identified by all four methods (Figure 7B). Functional enrichment analysis of these 52 candidate genes revealed significant enrichment in pathways including the cell cycle, neuroactive ligand-receptor interaction, and protein export (Figure 7C). GO enrichment analysis indicated significant enrichment for molecular functions such as chloride channel activity (Figure 7D).

### 3.7. Characterizing Genomic Adaptations to Altitude Environments Through SV Analysis

**Figure 8:**
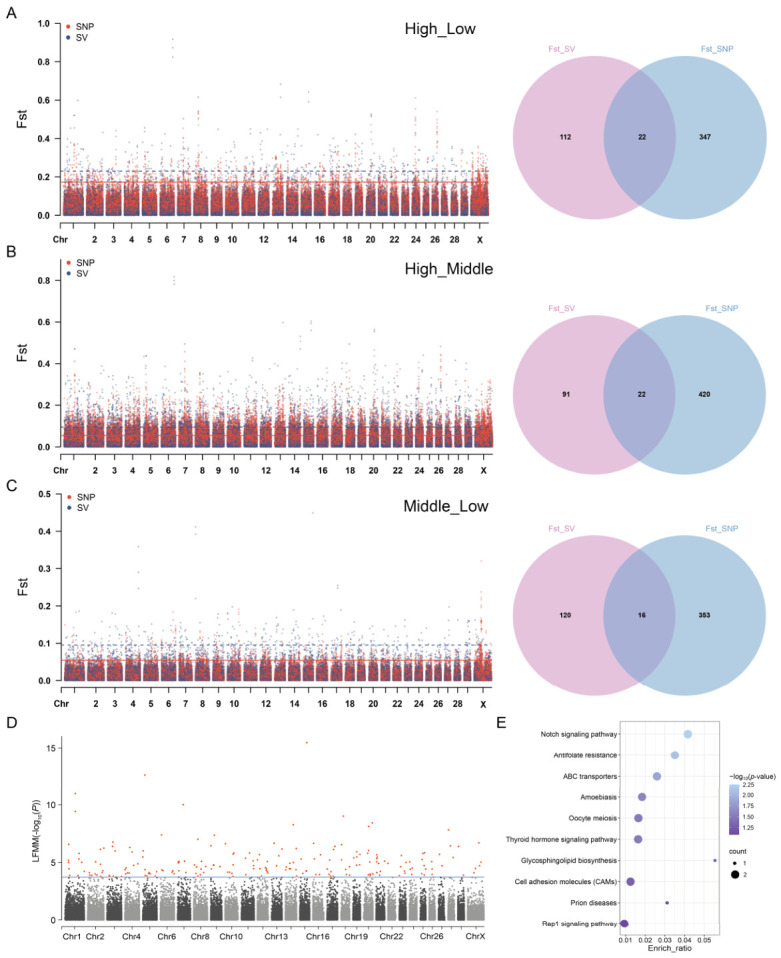
Genome-wide selection signatures identified through F_ST_ and LFMM tests based on SVs. (**A**) F_ST_ analysis between high-altitude and low-altitude populations. Red dots represent SNPs, with the red threshold line indicating the empirical top 1% SNP outlier cutoff (F_ST_ _SNP > 0.172803). Blue dots represent SVs, with the blue threshold line indicating the top 1% of structurally selected variants (F_ST_ _SV > 0.230043). The right-hand panel shows the overlap between genes identified from SV-based and SNP-based candidate windows. (**B**) F_ST_ analysis between high-altitude and middle-altitude populations. F_ST_ _SNP > 0.148412, F_ST_ _SV > 0.227004. (**C**) FST analysis between middle-altitude and low-altitude populations. F_ST_ _SNP > 0.0544561, F_ST_ _SV > 0.0953732. (**D**) Distribution of significance values for SV frequency correlations with altitude variables in the LFMM test. The horizontal dotted line indicates the threshold of top 1% −log10(*p*-adj). SVs with *p*-adj < 0.05 are considered candidate SVs significantly associated with altitude. (**E**) KEGG functional enrichment analysis of candidate genes identified by the GWEAS.

We conducted F_ST_ analysis using the SV dataset to compare goats from different altitude groups, defining the top 1% of genomic windows as candidate regions. Using low-altitude goat populations as the control, we detected 410 selection signals in the high-altitude group (annotated to 134 genes) and 358 signals in the middle-altitude group (annotated to 136 genes) (Figure 8A,C). With middle-altitude populations as the control, we identified 390 selection signals in high-altitude groups, corresponding to 113 annotated genes (Figure 8B). Across the three pairwise comparisons, 53 genes were consistently detected in two or more groups. KEGG enrichment analysis revealed significant enrichment of these genes in pathways including ABC transporters, cytokine-cytokine receptor interaction, platelet activation, neuroactive ligand-receptor interaction, and phototransduction, primarily associated with energy metabolism, angiogenesis, nervous system function, and immune regulation (Table S9). Notably, *ABCC4* gene, which has undergone strong selection in SNPs, harbors a DEL (Chr12: 18215191) that has been subject to intense selection in both the high-low and high-middle altitude subgroups.

To evaluate the consistency of selection signals across variant types, we compared F_ST_ results derived from SNPs and SVs. In the three comparison groups, 22, 22, and 16 genes, respectively, showed concurrent significant selection signals at both SNP and SV levels (Figure 8A–C, right). These overlapping genes (49 non-redundant genes, e.g., *ABCC4*, *LY9*, *PDE6A*) were significantly enriched in pathways related to fatty acid biosynthesis and energy metabolism, such as phenylalanine, tyrosine and tryptophan biosynthesis and phenylalanine metabolism (Figure S5). Interestingly, candidate genes were also significantly enriched in the phototransduction pathway, which is involved in visual signal transduction.

Additionally, we performed a genome-wide environmental association study (GWEAS) on goat populations using the latent factor mixed model (LFMM) incorporating altitude variables and SVs. We identified 204 altitude-associated SVs (adjusted *p*-value < 0.05), involving 63 non-redundant genes (Figure 8D and Figure S6). These genes are primarily implicated in angiogenesis and cardiovascular function (*KAT2B*, *PDGFC*, *PRKD1*, *CADM1*, *ABCC4*), nervous system development (*ASTN2*, *MDGA1*, *SHISA6*), cell cycle regulation (*MAD1L1*, *SGO1*), and immune function (*FYB1*, *RNF11*). KEGG enrichment analysis showed that these candidate genes were significantly enriched in pathways relevant to high-altitude adaptation, including Notch signaling pathway, ABC transporters, Thyroid hormone signaling pathway, and Cell adhesion molecules (CAMs) (Figure 8E).

### 3.8. Candidate Genes Underlying Altitude Adaptation in Goats

**Figure 9:**
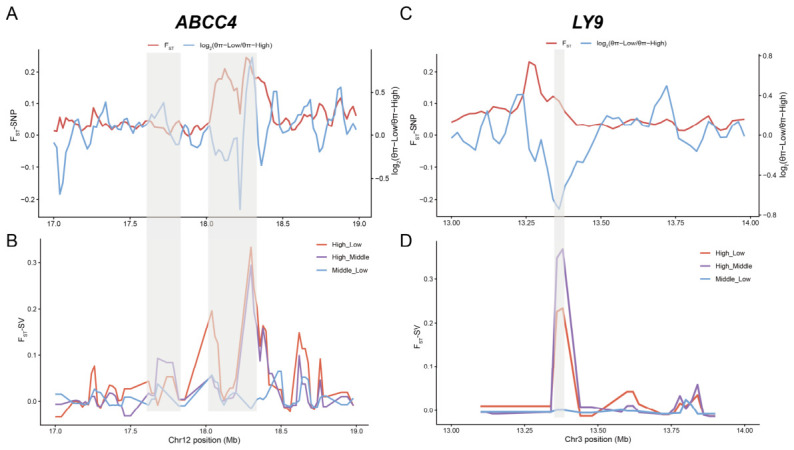
Selective sweep signals in the *ABCC4* and *LY9* genomic region. (**A**) Distribution of F_ST_ and θπ ratio signals around the *ABCC4* between high-altitude and low-altitude populations based on SNPs. Red curves show F_ST_ values; blue curves show log_2_(θπ) values. (**B**) Distribution of F_ST_ signals around the *ABCC4* based on SVs. (**C**) Log_2_(θπ ratios) and F_ST_ values around the *LY9* locus based on SNPs. (**D**) F_ST_ values around the *LY9* locus based on SVs. The gray shaded area indicates the genomic location of the *ABCC4* and *LY9*.

Integrative analysis across all selection scans and comparisons showed that *ABCC4* exhibited the most robust and consistent selection signals. Notably, we observed significantly elevated F_ST_ and reduced log_2_(θπ) values in the *ABCC4* genomic region (Figure 9A,B and Figure S7), consistent with a strong selective sweep. ATP-binding cassette sub-family C member 4 (*ABCC4*), a member of the ABC transporter family, has been demonstrated to be associated with immune system function in goats and contributes to environmental adaptation [51,52]. It is also implicated in the genetic domestication of sheep and goats [53,54]. Furthermore, *ABCC4* is involved in regulating platelet aggregation [55,56] and adipogenesis [57]. Studies have also shown a role for *ABCC4* in environmental adaptation via osmoregulation in pacific white shrimp [58].

Beyond *ABCC4*, which showed consistently strong selection signals across all methods, we observed heterogeneity in other selection signals depending on the analytical method and altitudinal comparison. For example, based on SNP data, genes such as *DSG4* (Figure S8), *SLC2A9* (Figure S9), and *RPS6* showed significant selection signals in particular altitude comparisons but did not show strong selection in the SV-based analysis. We also identified the *LY9* gene, which was under strong selection specifically in high-altitude groups but not in middle-altitude or low-altitude groups (Figure 9C,D). *LY9* has been reported to be involved in adaptive immune regulation [59]. However, because its signal was restricted to the high-altitude group, it may also reflect population-specific demographic history.

## 4. Discussion

In this study, we analyzed whole-genome resequencing data from 151 goats representing 17 Chinese indigenous breeds. These goats inhabit environments stratified as low-altitude (<1000 m), middle-altitude (1000–4000 m), and high-altitude (>4000 m) regions. We identified genome-wide SNPs and SVs and used these to elucidate the population genetic structure and genetic diversity. We also performed selective sweep analyses on both SNP and SV datasets to identify candidate variants associated with altitudinal adaptation. This work provides novel insights into goat adaptation and highlights the contribution of SVs to genomic architecture and adaptive variation.

### 4.1. Population Structure, Genetic Diversity, and SV Landscape Provide the Foundation for Altitude Adaptation

Population genetic structure analysis based on SNPs revealed a clear separation between high-altitude and low-altitude goat populations, indicating that altitude-related geography, together with breed history, contributed to the observed genetic differentiation. High-altitude goats (ZXB, CQ, LZ) formed a distinct cluster, consistent with their long-term habitation of the Qinghai–Tibetan Plateau (QTP) and exposure to strong selective pressures such as hypoxia, extreme cold, and intense ultraviolet (UV) radiation. In contrast, middle-altitude populations (notably WZMQ and ALS) exhibited higher genetic admixture. TreeMix analysis also detected gene flow events among these goat populations. In particular, TreeMix analysis revealed an absence of gene flow between Xizang goat breeds and other domestic breeds. This population isolation likely results from geographical barriers and extreme environmental conditions.

ROH analysis showed a higher proportion of ROH segments > 1 Mb in the AEBS, LN, and LZ populations (Figure 2A). This pattern likely resulted from recent inbreeding in these populations and further supported the effects of sustained artificial selection. High-altitude goat populations (ZXB, CQ, LZ) exhibited slower LD decay rates and higher genetic diversity, further suggesting they have experienced stronger selection pressures, likely driven by the extreme local environment (altitude > 4000 m). Other populations such as AEBS and LN showed the fastest LD decay, likely due to sustained artificial selection leading to reduced genetic diversity.

To maximize SV detection accuracy from short-read sequencing data, we employed five high-confidence callers (Delly, Lumpy, Dysgu, Manta, and Wham). Only SVs ≤ 1 Mb that were detected by at least three callers were retained for downstream analysis. This strategy improved the reliability of our SV dataset [60]. SV-based population structure was consistent with SNP-based results, indicating that our SV dataset effectively captured the population structure of the 17 breeds. However, compared to SNPs, admixture analysis based on SVs revealed finer and more complex patterns of population differentiation, particularly in the high-altitude populations. As the assumed number of ancestral populations increased, Xizang goats (ZXB, CQ, LZ) displayed discordant ancestral compositions. ZXB and CQ shared one ancestral component, while LZ formed a distinct component, suggesting distinct ancestral origins for goat populations within Xizang Autonomous Region. Furthermore, LD analysis between SVs and neighboring SNPs revealed that only 3.8% of SVs showed any LD (R^2^ > 0) with SNPs, with moderate-to-high LD (0.2 < R^2^) accounting for only 6.52% of these. These results indicate that SVs provide unique genetic information independent of SNPs.

In addition, we identified 975 SV hotspots across the goat genome, spanning approximately 373 Mb, which aligns with previous observations on SV enrichment patterns in livestock genomes [41]. To assess the potential role of SV hotspots in economically important traits, we compared these hotspot regions with goat QTL data from the Animal QTL Database. We observed overlaps between SV hotspots and QTLs for traits such as body height, body length, chest width, and milk fat percentage. This further indicates that SVs play a significant role in livestock domestication and the formation of economic traits [61].

### 4.2. Multi-Level Genomic Signatures Reveal Adaptive Strategies Across Altitudes

Adaptation refers to phenotypic and genetic changes driven by natural selection that enable organisms to better survive in their environments and transmit genetic information [62]. Animal adaptation to high-altitude environments primarily involves hypoxia response, resistance to intense UV radiation, immune modulation, and energy utilization [63,64]. Previous studies on altitudinal adaptation in goats have been limited by a narrow range of breeds, relatively low sequencing depth, and a predominant focus on comparing only high-altitude and low-altitude populations. Therefore, to systematically decipher the high-altitude adaptation mechanisms in goats, we performed pairwise genome-wide selection sweeps using SNPs across three altitude groups, identifying 71, 84, and 52 candidate genes. Notably, genes identified in the high-altitude versus low-altitude comparison were primarily enriched in pathways related to cardiovascular homeostasis and hypoxia adaptation (PI3K–Akt signaling pathway, mTOR signaling pathway), oxidative stress (ABC transporters, p53 signaling pathway), and energy metabolism (Carbon metabolism). In contrast, differentially selected genes from the middle-altitude versus low-altitude comparison were enriched in pathways involving neural regulation (Neuroactive ligand–receptor interaction, Glutamatergic synapse) and cell proliferation (Cell cycle). These results suggests that goats at middle-altitude may primarily adapt through neural regulation and cellular homeostasis adjustments. Conversely, high-altitude goats are exposed to hypoxia, intense UV radiation, and low temperatures, and their adaptations center primarily on cardiovascular regulation, oxidative stress response, and energy metabolism. Similar patterns were also observed in the high-altitude versus middle-altitude comparison.

The PI3K–Akt and mTOR signaling pathway have been closely associated with altitude adaptation in animals [65,66,67]. Hypoxia induces proangiogenic signals (VEGF/FGF) that activate receptor tyrosine kinases and trigger MAPK and PI3K–Akt cascades, thereby promoting angiogenesis and vasodilation [68]. This pathway is also a potential mechanism for crustacean adaptation to high-altitude environments [69]. Under hypoxic conditions, mTOR activity is suppressed, which is recognized as a crucial mechanism for maintaining cellular energy homeostasis. The hypoxia-inducible transcription factor (HIF) is a master regulator of hypoxic adaptation, and its translation is modulated by the mTOR signaling pathway [70]. This pathway promotes adaptation to hypoxia by regulating proliferation, apoptosis, and autophagy of coronary artery smooth muscle cells [71]. Neuroactive ligand signaling contributes to high-altitude adaptation by participating in physiological regulation under hypoxia in species such as yak [72] and mouse [73]. UV radiation is an important factor in the adaptation of high-altitude flora and fauna to their environment, influencing diverse biological processes. For example, maca plants grown at high altitudes show upregulation of glutathione metabolism under UV exposure, which enhances UV resistance [74]. Furthermore, glutathione metabolism pathway has been implicated in hypoxia adaptation in sheep [75], rabbits [76], and mice [77,78]. In summary, the pathways enriched for candidate genes identified from SNP-based selective sweep analyses are extensively involved in physiological processes such as hypoxia response, energy metabolism, and oxidative stress. These findings indicate that in high-altitude environments, organisms must cope with selective pressures from hypoxia, intense UV radiation, and low temperatures, and that these signaling pathways and candidate genes are crucial for maintaining homeostasis and normal physiological functions.

We also performed a genome-wide selective sweep analysis on the SV dataset using the F_ST_ method. The enrichment results for SV-derived candidate genes were similar to those from SNPs, primarily involving signaling pathways related to energy metabolism, angiogenesis, nervous system function, and immune regulation (Table S9). This further underscores the critical role of these signaling pathways in the altitudinal adaptation of goats. Additionally, we compared the F_ST_ results derived from SVs with those from SNPs. Across the three comparisons, a total of 49 non-redundant overlapping genes were identified. Among these, the *PDE6A* gene was significantly enriched in the phototransduction pathway. *PDE6A* is involved in the transmission and amplification of visual signals [79]. High-altitude regions experience intense UV radiation, which can damage DNA, induce oxidative stress, and also affect the visual system and its signal transduction processes (e.g., photoreception of UV wavelengths and retinal adaptation mechanisms) [80]. We hypothesize that *PDE6A* may influence adaptive adjustments in the visual system or photoreception mechanisms of high-altitude goats by modulating the phototransduction pathway. We propose that *PDE6A* may contribute to maintaining visual homeostasis and mitigating UV-induced oxidative damage in high-altitude goats.

Additionally, we conducted a GWEAS using the LFMM method using SV genotype data. This analysis identified 63 non-redundant genes (*p*-adj < 0.05), including *ABCC4*, *ABCC5*, *CDK14*, *SLC2A9*, *KCNH8*, *PRKD1*, and *TANK*, which were also detected in the F_ST_ analysis of SVs. These genes were significantly enriched in vascular-related pathways, such as the Notch signaling pathway (*KAT2B* and *MAML3*) and Cell adhesion molecules (CAMs) (*NRXN1* and *CADM1*). Notably, Angiogenesis and cardiovascular function are critical for high-altitude adaptation. The Notch signaling pathway is extensively involved in angiogenesis and cell differentiation, and it regulates vascular remodeling and tissue development under hypoxic conditions [81,82,83]. In rats, miR-203a-3p can inhibit the activation of the Notch signaling pathway, thereby limiting the angiogenic capacity of pulmonary vascular endothelial cells [84]. It also regulates hypoxia tolerance mechanisms in Drosophila [85]. Research indicates that the hypoxic environment at high altitudes can damage the intestinal mucosal barrier, leading to gastrointestinal mucosal dysfunction. Activation of Notch signaling pathway may represent a mechanism underlying the reduction in mucin-2 and dysfunction of the intestinal mucosal barrier [86]. Cell adhesion molecules (CAMs) play key roles in cell-to-cell communication and maintaining vascular barrier integrity [87]. These selective sweep results indicate that high-altitude adaptation in goats is not governed by a single type of genetic variation, it is a combined effect of SNPs and SVs, which contribute to organismal adaptation at different genetic variation levels. Thus, high-altitude adaptation is a physiological process driven by multi-gene and multi-level regulation.

### 4.3. Functional Implications of Candidate Genes

Because the altitude groups consisted of different breeds from distinct geographic regions, altitude-related effects may be partially confounded by breed history and demographic processes. Although population structure analyses and LFMM-based environmental association analysis were used to reduce this bias, the genes identified here should be interpreted as putative altitude-associated candidates rather than confirmed causal genes. We therefore prioritized genes according to recurrence across pairwise comparisons, support from multiple selection-scan methods, concordance between SNP-based and SV-based analyses, and functional relevance to altitude-related physiology.

In this study, *ABCC4* showed recurrent selection signals across pairwise altitude comparisons (high vs. low, high vs. middle, and middle vs. low) and in analyses based on both SNPs and SVs. Unlike canonical hypoxia-response genes such as *EPAS1* [88] or *EGLN1* [89], *ABCC4* is not a classical oxygen-sensing gene. *ABCC4* encodes an ATP-binding cassette transporter involved in biological processes such as transmembrane transport [90], immune regulation [51], and platelet function modulation [55]. Previous goat genomic studies have also implicated *ABCC4* in domestication and cashmere-related selection [91], and recent T2T goat genome analysis reported strong selection signals involving tandemly repeated *ABCC4* genes in cashmere goats, with potential relevance to keratinocyte differentiation and stratum corneum keratinization [53]. Therefore, our *ABCC4* signal may reflect an indirect adaptive route involving immune balance, vascular or platelet-related regulation, and cellular homeostasis under chronic altitude-related stress. *ABCC4* should be viewed as a high-priority hypothesis rather than a confirmed hypoxia-adaptation gene.

In addition to *ABCC4*, we also identified other selected genes recurrently detected across different analytical methods, including *RPS6*, *DSG4*, and *LY9*. The recurrent selection signal at *RPS6* further highlights the importance of metabolic regulation in goat high-altitude adaptation. As a downstream regulator of the mTOR pathway, *RPS6* integrates nutrient sensing and cellular energy status under hypoxia [92]. Under hypoxic conditions, energy stress can regulate mTOR signaling and downstream *RPS6* through the AMPK/TSC2/Rheb/mTOR pathway. The observed reduction in *RPS6* signaling under high aerobic demands, as reported in canines [93], suggests that similar mechanisms may operate in goats to fine-tune mitochondrial function and protein synthesis under chronic cold and hypoxic stress. Moreover, reduced *RPS6* signaling can increase mitochondrial protein synthesis rates, thereby helping to meet elevated protein demands under high aerobic conditions in canines. Thus, selection involving *RPS6* may reflect modulation of protein synthesis, cell growth, and energy conservation under chronic environmental stress rather than direct oxygen sensing. To our knowledge, *RPS6* has not been repeatedly reported as a major candidate gene in previous high-altitude goat selection studies. Nevertheless, our multi-layered genomic scans (integrating SNPs and SVs) implicate *RPS6* as a potentially novel effector of environmental adaptation in goats.

*DSG4* is a member of the desmoglein family within the cadherin superfamily, encoding a transmembrane glycoprotein that plays a critical role in cell–cell adhesion and hair follicle morphogenesis [94]. Previous studies in Chinese cashmere goats identified *DSG3* variants that differentiated high-altitude and low-altitude populations, and comparative genomic analysis of Xizang domestic mammals further reported that the *DSG2*–*DSG3*–*DSG4* gene cluster was under selection in Xizang goats [95,96]. In addition, *DSG4* has also been reported among candidate regions associated with adaptation to cold environments in high-altitude livestock [97]. Therefore, our *DSG4* signal is consistent with earlier evidence implicating this genomic region in high-altitude goat adaptation. However, the biological meaning of *DSG4* may not be limited to hypoxia response. *DSG4* is involved in epithelial adhesion, hair follicle differentiation, and coat-related traits [98,99], and goat studies have linked *DSG4* to coat color and cashmere hair follicle regulation [94]. Given that high-altitude environments combine hypoxia with cold temperature and intense ultraviolet radiation, selection at *DSG4* may reflect integumentary adaptation, skin barrier maintenance, hair follicle regulation, or thermal insulation, rather than hypoxia adaptation alone. *LY9* plays a critical role in adaptive immune regulation in animals [59]. In mice, *LY9* deficiency leads to systemic autoimmune features and elicits cell-intrinsic protective mechanisms that help prevent tolerance breakdown [100]. This further underscores the importance of immune regulation in environmental adaptation. In addition, the *LY9* signal in our study was mainly observed in the high-altitude group, which had a smaller sample size and restricted geographical distribution. Therefore, *LY9* may represent a population-specific immune-related candidate, but founder effects, genetic drift, or breed history cannot be excluded.

Although this study identified candidate genes and pathways potentially associated with goat altitude adaptation based on whole-genome resequencing and multiple SNP- and SV-based selection scans, several limitations should be acknowledged. First, unequal sample sizes among altitude groups, particularly the smaller high-altitude group, may affect allele-frequency-based statistics. Second, the middle-altitude group spans a wide elevation range and may therefore represent heterogeneous ecological conditions and should be interpreted as an intermediate altitude-related category rather than a physiologically uniform population. Third, although several candidate genes (e.g., *ABCC4*) showed recurrent signals and functional relevance to altitude-related biological processes, no expression or functional validation was performed in this study. In addition, SVs were inferred from short-read sequencing data using a multi-caller strategy. While this approach improves reliability and reduces method-specific false positives, short-read sequencing is inherently less accurate for detecting INSs than DELs or DUPs, particularly in repetitive genomic regions. Consequently, INS calls are expected to exhibit a higher false-positive risk than other SV types, and some INS events reported here may therefore represent residual false positives despite the use of five independent callers and stringent consensus filtering. Furthermore, the requirement for concordant signals across multiple selection-scan methods is a conservative strategy that reduces false positives but may also lead to false negatives by excluding loci detected by only one or two methods. Taken together, these genes should be interpreted as putative candidates rather than confirmed causal genes for altitude adaptation. Future studies incorporating larger and more balanced sampling, finer altitude stratification, long-read sequencing or PCR-based validation of structural variants, and multi-omics or functional experiments will be needed to further improve the robustness of these findings.

## 5. Conclusions

This study elucidates the genetic basis of high-altitude adaptation in goats by analyzing whole-genome resequencing data from 151 individuals across three altitudinal gradients. We analyzed SNP data to infer population structure, genetic diversity, and gene flow, providing a comprehensive understanding of the phylogenetic relationships among domestic goats. Concurrently, we identified 53,500 high-quality SVs using five tools, which supported the population structure inferred from SNPs. Moreover, by assessing LD with SNPs, we demonstrated that goat SVs represent genetic information independent of SNPs. Selective sweep analyses using multiple methods on both SNPs and SVs revealed that high-altitude goats are under selection pressure in pathways related to energy metabolism, oxidative stress, and angiogenesis. We identified several candidate genes with putative key roles in adaptation, including *ABCC4*, *LY9*, *RPS6*, *DSG4*. These genes play crucial roles in various physiological processes, including hypoxia adaptation, cellular homeostasis, and angiogenesis, collectively supporting altitude adaptation in goats under hypoxic, UV, and cold. Notably, *ABCC4* exhibited recurrent selection signals in both SNP and SV analyses, suggesting that it may represent a strong putative candidate gene associated with altitude adaptation in goats. In summary, this study provides genomic evidence for candidate genes associated with altitude adaptation in goats from a multi-level perspective integrating SNPs and SVs, providing novel theoretical insights into livestock adaptive evolution.

## Data Availability

All data used for evaluating the conclusions in the paper are presented in the paper and the Supplementary Materials. The raw data have been deposited in the National Center for Biotechnology Information (NCBI) Sequence Read Archive (SRA) database under accession PRJNA1104411.

## Supplementary Materials

The following supporting information can be downloaded at: https://www.mdpi.com/article/10.3390/ani16142177/s1, Figure S1. PCA of 151 goat individuals based on SNP data; Figure S2. Population splits, migration events, and residual fit inferred by TreeMix among 17 Chinese goat breeds and *Capra ibex*; Figure S3. Genomic annotation distribution of SVs across different functional regions; Figure S4. Length distribution of SVs ranging from 50 to 500 bp; Figure S5. KEGG pathway enrichment analysis of candidate genes identified from overlapping SNP and SV selective signals; Figure S6. Determination of optimal K value for LFMM analysis; Figure S7. Selective sweep signals at the *ABCC4* locus across three altitude pairwise comparisons; Figure S8. Selective sweep signals in the *DSG4* genomic region; Figure S9. Selective sweep signals in the *SLC2A9* genomic region; Table S1. Information of 151 goat individuals aligned to goat genome; Table S2. ROH-based inbreeding coefficients of 17 goat breeds; Table S3. The expected heterozygosity and observed heterozygosity of each breed; Table S4. Number of SVs in goat populations at different altitudes; Table S5. Frequency distribution of SVs across different minor allele frequency; Table S6. Transposable element annotation of SVs; Table S7. The SV hotspot regions overlapping with QTL regions in goats; Table S8. Number of structural variation breakpoints in telomeric and non-telomeric regions of the goat genome; Table S9. KEGG pathway enrichment results for selection genes.

## Abbreviations

The following abbreviations are used in this manuscript:

SV	Structural Variation
PCA	Principal component analysis
*H*o	Observed heterozygosity
*H*e	Expected heterozygosity
ROH	Run of Homozygosity
F_ROH_	Inbreeding coefficient
QTL	Quantitative Trait Loci
F_ST_	The pairwise fixation index
XP-CLR	The cross population composite likelihood ratio
XP-EHH	The cross-population extended haplotype homozygosity
GWEAS	Genome-wide environmental association study
LFMM	Latent Factor Mixed Model
KEGG	Kyoto Encyclopedia of Genes and Genomes
GO	Gene Ontology
