# Supplementary material for: Integrated SNP and SV Analyses Reveal Genetic Mechanisms Underlying High-Altitude Adaptation in Goats
Source: Animals (Basel). 2026 Jul 13;16(14):2177. doi: 10.3390/ani16142177 (PMC13406086; doi:10.3390/ani16142177)
Supplement: Supplementary file 1 [file animals-16-02177-s001.zip › Supplementary figures.pdf]

Supplementary Files

File 1: Supplementary Figures

Figure S1. PCA of 151 goat individuals based on SNP data.

Figure S2. Population splits, migration events, and residual fit inferred by TreeMix among 17 Chinese goat breeds and *Capra ibex*.

Figure S3. Genomic annotation distribution of SVs across different functional regions..

Figure S4. Length distribution of SVs ranging from 50 to 500 bp.

Figure S5. KEGG pathway enrichment analysis of candidate genes identified from overlapping SNP and SV selective signals.

Figure S6. Determination of optimal K value for LFMM analysis.

Figure S7. Selective sweep signals at the *ABCC4* locus across three altitude pairwise comparisons.

Figure S8. Selective sweep signals in the *DSG4* genomic region.

Figure S9. Selective sweep signals in the *SLC2A9* genomic region.

File 2: Supplementary Tables

Table S1. Information of 151 goat individuals aligned to goat genome.

Table S2. ROH-based inbreeding coefficients of 17 goat breeds.

Table S3. The expected heterozygosity and observed heterozygosity of each breed.

Table S4. Number of SVs in goat populations at different altitudes.

Table S5. Frequency distribution of SVs across different minor allele frequency.

Table S6. Transposable element annotation of SVs.

Table S7. The SV hotspot regions overlapping with QTL regions in goats.

Table S8. Number of structural variation breakpoints in telomeric and non-telomeric regions of the goat genome.

Table S9. KEGG pathway enrichment results for selection genes.

Supplementary Figures:

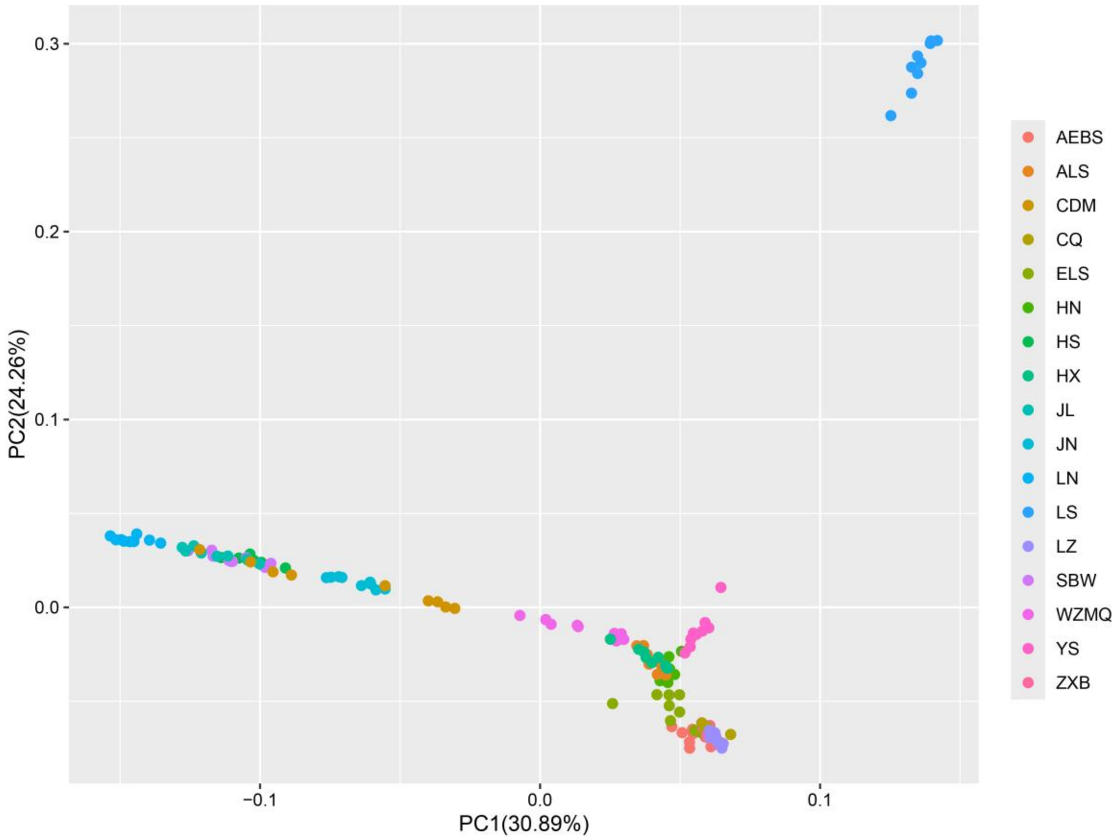

Figure S1. PCA of 151 goat individuals based on SNP data.

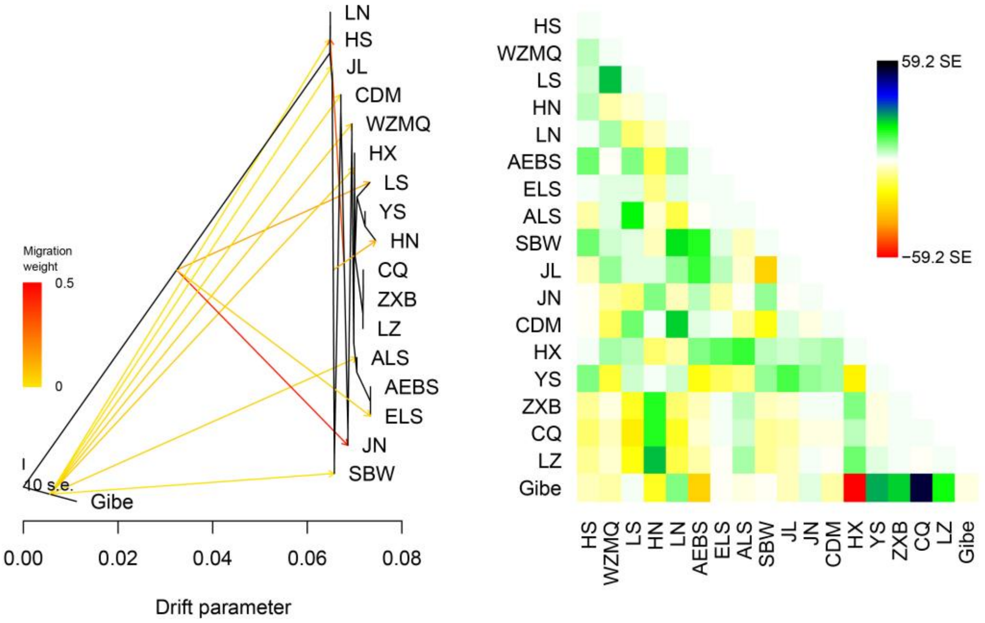

Figure S2. Population splits, migration events, and residual fit inferred by TreeMix among 17 Chinese goat breeds and *Capra ibex*.

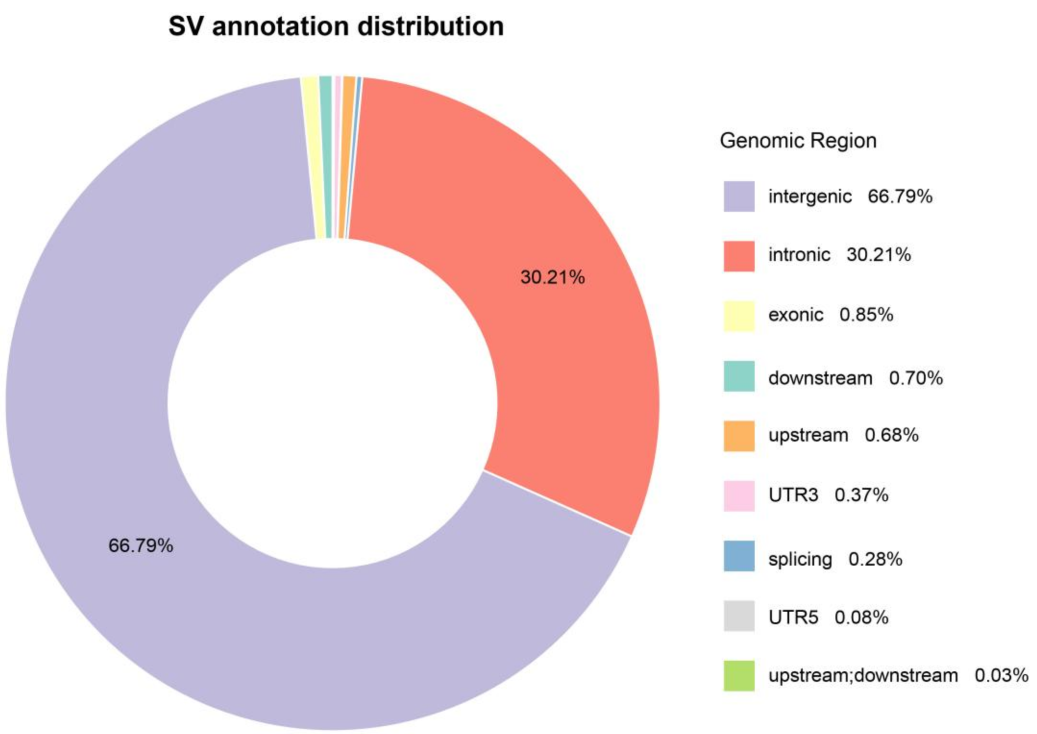

Figure S3. Genomic annotation distribution of SVs across different functional regions.

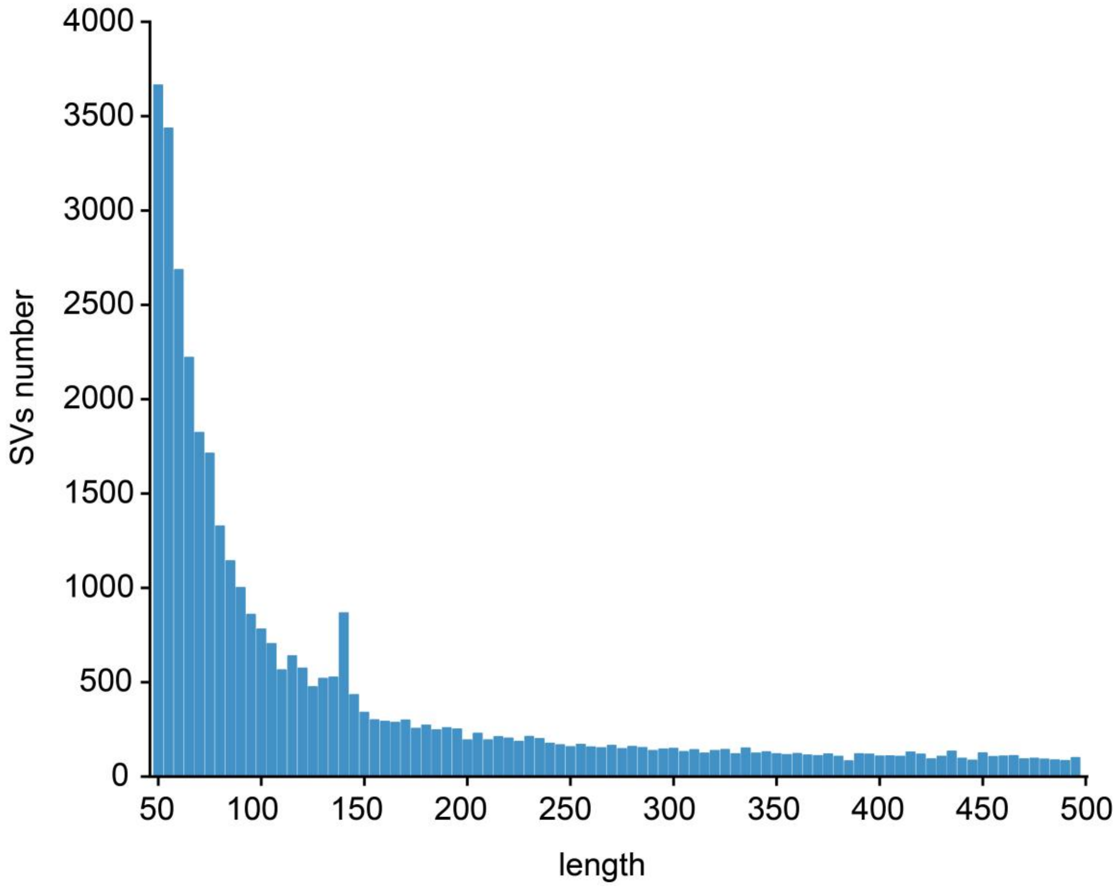

Figure S4. Length distribution of SVs ranging from 50 to 500 bp.

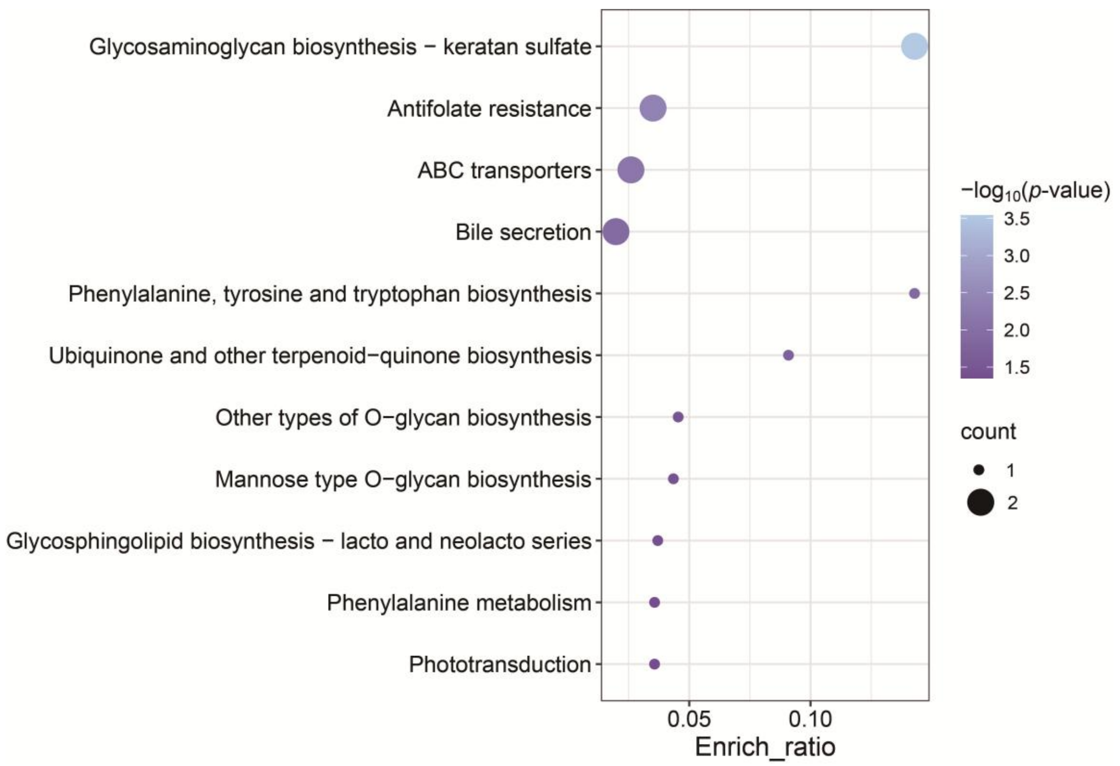

Figure S5. KEGG pathway enrichment analysis of candidate genes identified from overlapping SNP and SV selective signals.

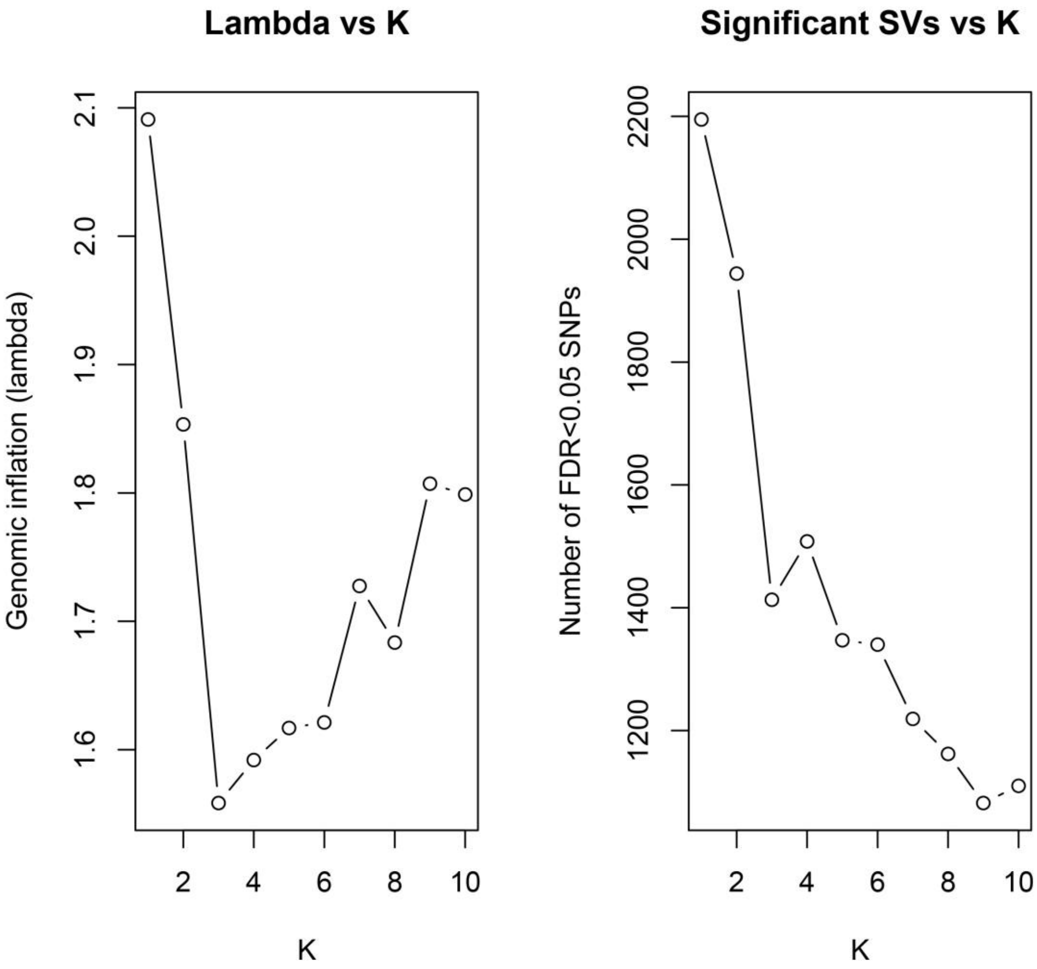

Figure S6. Determination of optimal K value for LFMM analysis.

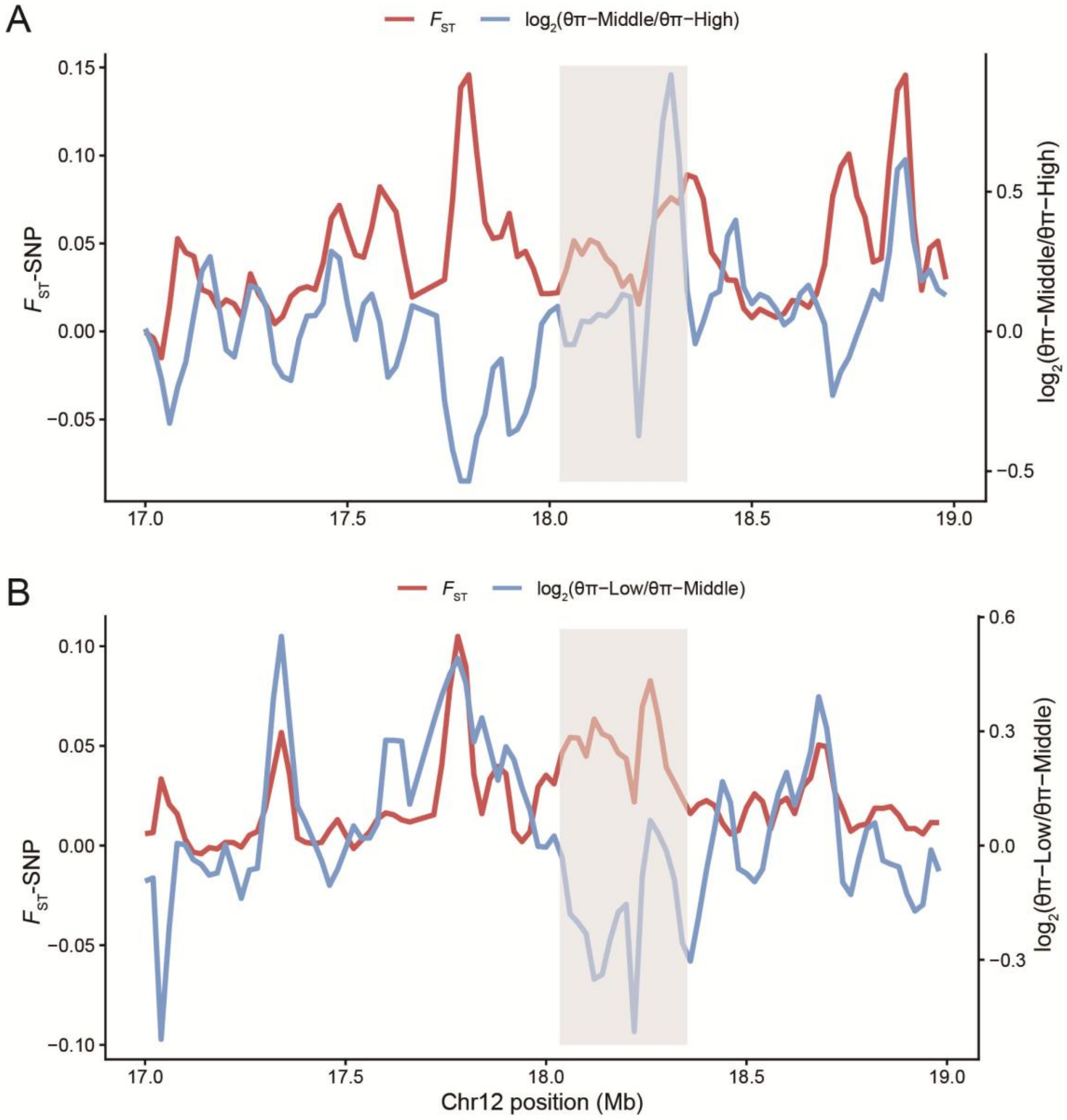

Figure S7. Selective sweep signals at the *ABCC4* locus across three altitude pairwise comparisons.

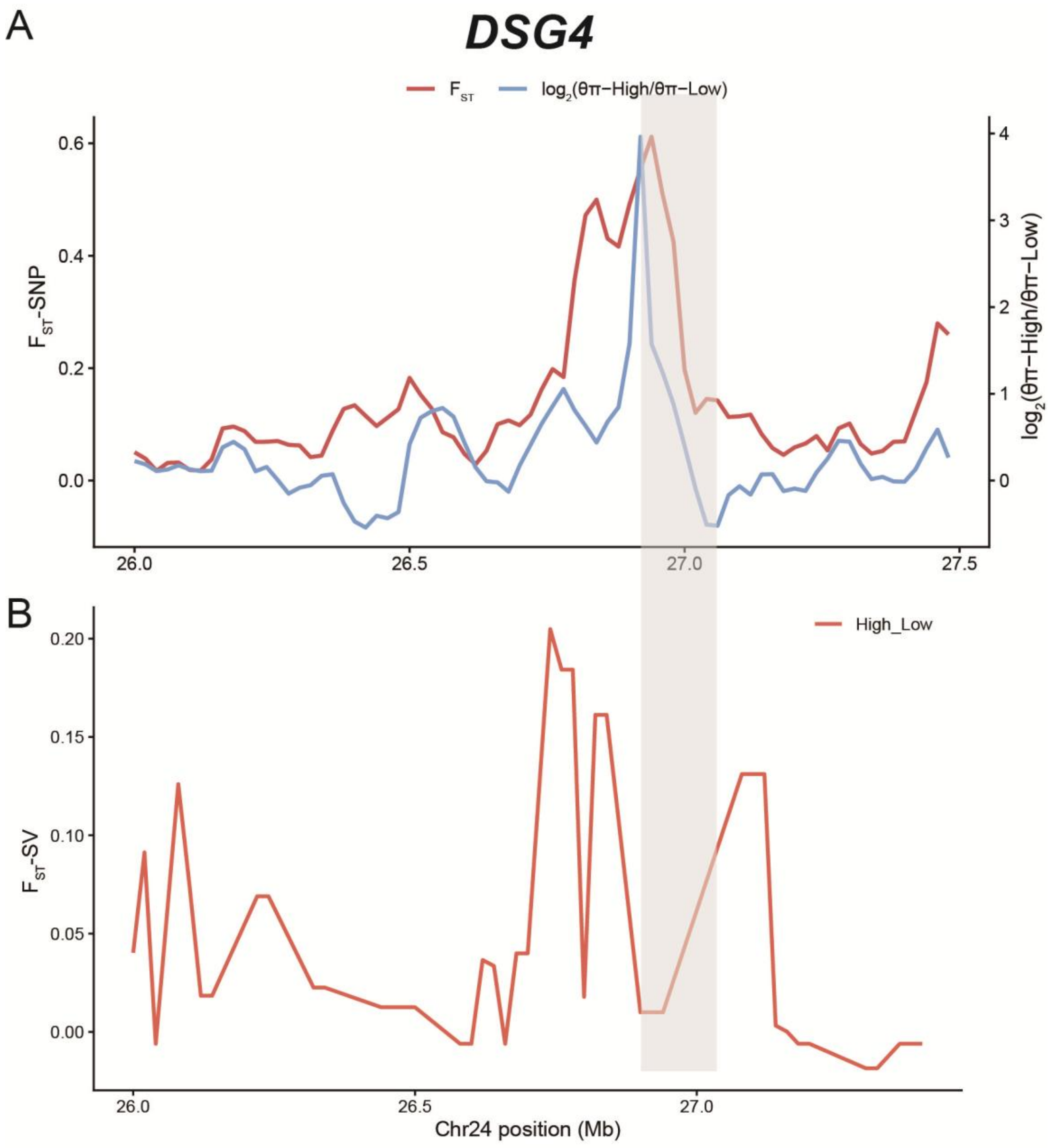

Figure S8. Selective sweep signals in the *DSG4* genomic region.

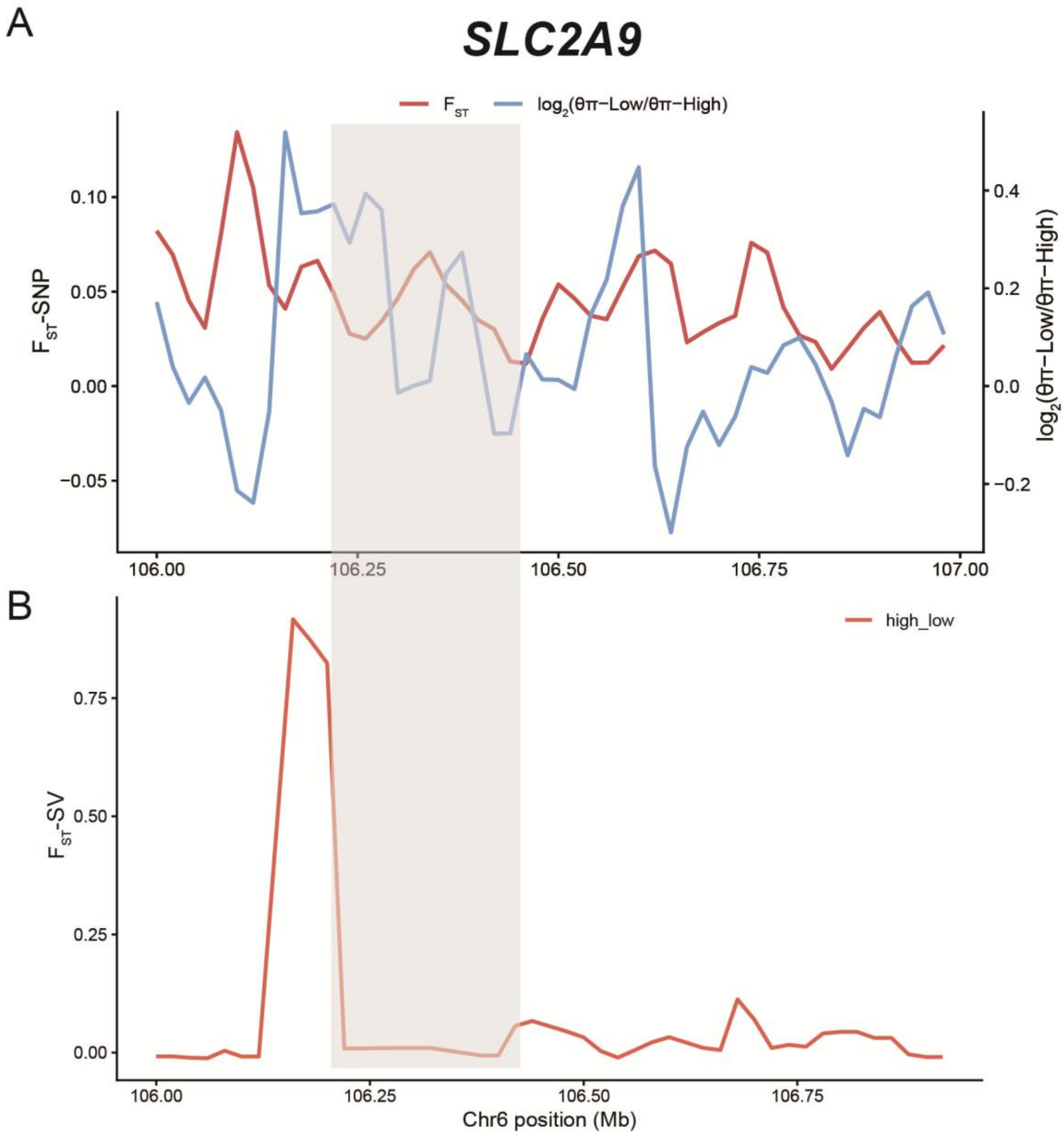

Figure S9. Selective sweep signals in the *SLC2A9* genomic region.
